# Two-Level Scheme for Identification of the Relaxation Time Spectrum Using Stress Relaxation Test Data with the Optimal Choice of the Time-Scale Factor

**DOI:** 10.3390/ma16093565

**Published:** 2023-05-06

**Authors:** Anna Stankiewicz

**Affiliations:** Department of Technology Fundamentals, Faculty of Production Engineering, University of Life Sciences in Lublin, 20-612 Lublin, Poland; anna.m.stankiewicz@gmail.com

**Keywords:** viscoelasticity, relaxation time spectrum, linear relaxation modulus, hierarchical identification algorithm, regularized least-squares identification, time-scale factor optimal selection, modified Bessel functions of the second kind, singular value decomposition

## Abstract

The viscoelastic relaxation spectrum is vital for constitutive models and for insight into the mechanical properties of materials, since, from the relaxation spectrum, other material functions used to describe rheological properties can be uniquely determined. The spectrum is not directly accessible via measurement and must be recovered from relaxation stress or oscillatory shear data. This paper deals with the problem of the recovery of the relaxation time spectrum of linear viscoelastic material from discrete-time noise-corrupted measurements of a relaxation modulus obtained in the stress relaxation test. A two-level identification scheme is proposed. In the lower level, the regularized least-square identification combined with generalized cross-validation is used to find the optimal model with an arbitrary time-scale factor. Next, in the upper level, the optimal time-scale factor is determined to provide the best fit of the relaxation modulus to experiment data. The relaxation time spectrum is approximated by a finite series of power–exponential basis functions. The related model of the relaxation modulus is proved to be given by compact analytical formulas as the products of power of time and the modified Bessel functions of the second kind. The proposed approach merges the technique of an expansion of a function into a series of independent basis functions with the least-squares regularized identification and the optimal choice of the time-scale factor. Optimality conditions, approximation error, convergence, noise robustness and model smoothness are studied analytically. Applicability ranges are numerically examined. These studies have proved that using a developed model and algorithm, it is possible to determine the relaxation spectrum model for a wide class of viscoelastic materials. The model is smoothed and noise robust; small model errors are obtained for the optimal time-scale factors. The complete scheme of the hierarchical computations is outlined, which can be easily implemented in available computing environments.

## 1. Introduction

Numerous mathematical rheological models are used to describe the mechanical properties of viscoelastic materials [1,2,3]. The Maxwell and Kelvin–Voight models are, probably, the best-known rheological models. However, deeper insight into the complex behavior of viscoelastic materials is provided by the relaxation spectrum [2,4,5]. The relaxation spectrum is vital for constitutive models and for insight into the properties of a material, since, from the relaxation spectrum, other material functions used to describe rheological properties can be uniquely determined [4,6,7]. It is commonly used to describe, analyze, compare, and improve the mechanical properties of polymers [4,8,9], concrete [10], asphalt [11], rubber [12], wood [13], glass [14], dough [15], polymeric textile materials [16], geophysics [17] and biological materials [18]. The spectrum is not directly measurable; therefore, it must be recovered from oscillatory shear or stress relaxation data [2,5]. A number of different methods and algorithms have been proposed during the last five decades for the recovery of the relaxation spectrum of a viscoelastic material from oscillatory shear data. These works, using different models, approaches and computational techniques, have included contributions by Baumgaertel and Winter [19], Honerkamp and Weese [20], Malkin [21], Malkin et al. [22], Stadler and Bailly [23], Davis and Goulding [24], Davis et al. [25] and Cho [26]. These studies, but also many others, have created new directions of research on discrete and continuous relaxation spectra identification based on dynamic moduli data, and have been conducted ever since [8,11,27].

However, a classical way of studying viscoelasticity is also the stress relaxation test, where time-dependent shear stress is studied for the step increase in strain [1,2,3,28]. For some materials, for example, highly hydrated biological plants, the stress relaxation test is much easier to perform and is more suitable than dynamic oscillatory shear tests [3,29]. There are only a few papers that deal with spectrum determination from time measurements of the relaxation modulus. Additionally, some of them only address specific materials. The first works came from the turn of the 1940s and 1950s. Alfrey and Doty [30] proposed a simple differential model, being, in fact, the first-order Post–Widder formula [31] for the inverse Laplace transform; for details, see the newer edition [32]. Ter Harr [33] approximated the spectrum of relaxation frequencies by the modulus multiplied by time inverse of the relaxation frequency, which can be thought as the Post–Widder inversion formula of the zero order. After many years, Bažant and Yunping [34] and Goangseup and Bažant [35] introduced a two-stage approach of approximating the stress (or retardation) data via multiple differentiable models of the relaxation modulus (creep compliance) and, next, by applying the Post–Widder formula to designate the related relaxation (retardation) spectrum model. The effectiveness of this approach depends, among other aspects, on the function applied to approximate the relaxation modulus. In [34], a logarithmic–exponential model of the relaxation modulus is proposed, for which the authors state the third-order Post–Widder approximation to be satisfactory.

The relaxation spectrum modeling based on the known pairs of Laplace transforms was initiated by Macey [36], who described the relaxation modulus of viscoelastic ceramic material by the modified Bessel function of the second kind and zero order, which corresponds to the exponential–hyperbolic model of the spectrum. To describe the mechanical properties of polyisobutylene, Sips [37] introduced a simple relaxation spectrum model given by the difference of two exponential functions and a related logarithmic model of the modulus. This model was augmented to consider a long-term modulus by Yamamoto [38] and applied to test the rheological properties of the plant cell wall. 

Both the algorithms based on the Post–Widder formula and those using the pairs of Laplace transforms assumed rather narrow classes of models. Thus, the scope of their effective applicability is limited to similar, not much wider classes of ‘real’ relaxation characteristics. A wider range of applicability has been offered by the models based on the expansion of an unknown spectrum into a series of basis functions (or polynomials) forming a complete basis in a function space, for example, in the space of real-valued square-integrable functions. Stankiewicz [39] and Stankiewicz and Gołacki [18] derived algorithms of the optimal regularized identification of relaxation and retardation spectra in the classes of models generated by different special functions, where various rules were applied for the choice of regularization parameters. 

However, articles [18,39] were based on such a definition of the relaxation spectrum, according to which, the modulus was directly given by the Laplace integral of the spectrum. This spectrum definition is not often used in the literature. Therefore, computationally efficient algorithms to determine the relaxation spectrum applied to time measurements of the relaxation modulus are still desirable. Recently, for the dominant definition of the relaxation spectrum in the literature, a class of algorithms for relaxation spectrum recovery, which combines the technique of an expansion of a function into a series in an orthonormal basis with the least-squares regularized identification, has been derived by Stankiewicz [40]. Legendre, Laguerre, Chebyshev and Hermite functions were used as the basis functions for the spectrum model. In [40], the problem of determining the spectrum of relaxation frequencies was considered. The complementary problem of determining the spectrum of relaxation times based on the stress relaxation data is both analytically and numerically more difficult than the determination of the spectrum of relaxation frequencies, because the relaxation time occurs in the denominator of the exponential function in the spectrum definitional formula. This very task is the subject of this article. Previous studies [18,39,40] have suggested that by selecting an appropriate time-scale factor, a better fit of the model to the measurement data can be obtained. 

The objective of the present paper was to develop a model and an identification algorithm for the determination of the continuous relaxation time spectrum based on discrete-time measurements of the relaxation modulus, which, taking into account the ill-posedness of the original problem of the spectrum recovery and the idea of the optimal selection of the time-scale factor, will provide: (a) good approximation of the relaxation spectrum and modulus also due to the best choice of the time-scale factor; (b) smoothness of the spectrum fluctuations, even for noise-corrupted measurements; (c) noise robustness; (d) applicability to a wide range of viscoelastic materials; (e) ease of the implementation of the model and identification algorithm in available computing packages. The idea of the optimal choice of time-scale factor is used here for the first time in the context of relaxation spectrum identification. The goal of this work was the synthesis of the respective model and identification scheme and the analysis of their properties. A further purpose was the numerical verification of the model and algorithm for double-mode Gauss-like distribution used to describe the viscoelastic properties of various materials, in particular different polymers, wood and glass. 

A new hierarchical algorithm of noise robust approximation of the continuous spectrum of relaxation times by finite series of power–exponential basis functions is proposed. The components of the relaxation modulus model are given by compact analytical formulas described by the product of power of time and the modified Bessel function of the second kind. The main properties of the basis functions of relaxation spectrum and modulus models have been studied; positive definiteness, upper bounds, monotonicity and asymptotic properties have been examined. Ranges of applicability for different time-scale factors are determined. Since the problem of relaxation spectrum identification is an ill-posed inverse problem, the regularized least-squares identification technique is applied combined with generalized cross-validation to guarantee the stability of the scheme. The quadratic identification index refers to the measured relaxation modulus. The task of determining the best “regularized” model is solved at the lower level of the identification scheme. The appropriate choice of the time-scale factor on the upper level of the scheme ensures the best fit of the relaxation modulus to experimental data. 

The optimality conditions are derived both for the identification problem solved at the lower level and the task of the optimal choice of the time-scale factor at the upper level of the scheme. It is proved that the smoothness of the vector of the optimal model parameters implies smoothness of the fluctuations of the relaxation spectrum model. A direct formula and upper and lower bounds for the square integral norm of the smoothed spectrum model are derived. The accuracy of the spectrum model for noisy measurements of the relaxation modulus is studied, and the linear convergence to the model that we would obtain for the noise-free measurements is proved. 

To design a numerical algorithm based on the scheme, the computations should be arranged hierarchically in a two-level scheme, i.e., for each iteration of the minimization procedure at the upper level, the whole numerical procedure must be realized for the lower-level task. The complete computational procedure for determining the best model is described. The singular value decomposition technique is applied to simplify the algebraic computations. The identification scheme can be easily implemented in available computing environments. The numerical studies, especially the strong smoothing of the relaxation spectrum models in the example of a double-mode Gauss-like spectrum, with a simultaneous very good fit to the experimental data of the relaxation modulus models, suggests the need to explore the applicability of the regularized weighted least-squares [41] in the lower-level identification task. This will be the subject of further work. 

In Appendix A, the proofs and derivations of some mathematical formulas are given. Some tables have been moved to Appendix B to increase the clarity of the article.

## 2. Materials and Methods

### 2.1. Relaxation Time Spectrum

The uniaxial, non-aging and isothermal stress–strain equation for a linear viscoelastic material is represented by a Boltzmann superposition integral [2]:

(1) $$\sigma \left(t\right)=\int_{-\infty }^{t}G\left(t-u\right)\dot{\varepsilon }\left(u\right)du,$$

where u is the past time variable in the range −∞ to the present time t. In Equation (1), variables σ(t) and ε(t) denote, respectively, the stress and strain at time t, and G(t) is the linear relaxation modulus. Modulus G(t) is given by [2,5]:

(2) $$G\left(t\right)=\int_{0}^{\infty }\frac{H\left(\tau \right)}{\tau }e^{-t/\tau }d\tau ,$$

where H(τ) characterizes the distributions of relaxation times τ. The continuous relaxation spectrum H(τ) is a generalization of the discrete Maxwell spectrum [2,5] to a continuous function of the relaxation times τ. 

The problem of relaxation spectrum identification is the problem of solving a system of Fredholm integral equations of the first kind (2) obtained for discrete-time measurement data. In this paper, time measurements of the relaxation modulus are considered. This problem is ill-posed in the Hadamard sense [42], i.e., small changes in measured relaxation modulus can lead to arbitrarily large changes in the determined relaxation spectrum. As a remedy, some reductions in the set of admissible solutions or the appropriate regularization of the original problem are used. Both the techniques are applied here. 

### 2.2. Models

Assume that $H\left(\tau \right)\in L^{2}(0,\infty )$, where $L^{2}(0,\infty )$ is the space of real-valued square-integrable functions on the interval (0,∞). It is known that the set of the linearly independent functions $\left\{e^{-\alpha \tau },\tau e^{-\alpha \tau },\tau^{2}e^{-\alpha \tau },\ldots \right\}$ form a basis of the space $L^{2}(0,\infty )$ [43] (p. 125); here, α is a positive time-scaling factor. Since the maximum

$$\underset{\tau \geq 0}{max}\;{\bar{h}}_{k}\left(\tau ,\alpha \right)={\left(\frac{k}{\alpha }\right)}^{k}e^{-k}$$

of the function ${\bar{h}}_{k}\left(\tau ,\alpha \right)=\tau^{k}e^{-\alpha \tau }$ grows rapidly with k, it is convenient to expand the relaxation spectrum into a series of scaled basis functions

(3) $$h_{k}\left(\tau ,\alpha \right)={\left(\frac{\alpha \tau }{k}\right)}^{k}e^{-\alpha \tau +k},\;k=1,2,\ldots ,$$

with the first function

(4) $$h_{0}\left(\tau ,\alpha \right)=e^{-\alpha \tau },$$

as follows:

(5) $$H\left(\tau \right)=\sum_{k=0}^{\infty }g_{k}h_{k}\left(\tau ,\alpha \right),$$

where $g_{k}$ are real model coefficients. 

It is practical to replace the infinite summation in Equation (5) with a finite one of K first terms, i.e., to approximate the spectrum H(τ) by a model of the form

(6) $$H_{K}\left(\tau ,\alpha \right)=\sum_{k=0}^{K-1}g_{k}h_{k}\left(\tau ,\alpha \right),$$

where the lower index is the number of model summands. Then, according to (2), the respective model of the relaxation modulus is described by:

(7) $$G_{K}\left(t,\alpha \right)=\int_{0}^{\infty }\frac{H_{K}\left(\tau ,\alpha \right)}{\tau }e^{-t/\tau }d\tau =\sum_{k=0}^{K-1}g_{k}\phi_{k}\left(t,\alpha \right),$$

where the functions

(8) $$\phi_{k}\left(t,\alpha \right)=\int_{0}^{\infty }\frac{h_{k}\left(\tau ,\alpha \right)}{\tau }e^{-t/\tau }d\tau .$$

For computational purposes, function $h_{0}\left(\tau ,\alpha \right)$ is defined by (4). However, since most mathematicians agreed that $0^{0}=1$ [44], the general Formula (3) can also be applied in further analysis for k=0.

The basis functions $\phi_{k}\left(t,\alpha \right)$ (8) of the modulus model (7) are given by a compact analytical formula specified by the following theorem proved in Appendix A.1.

**Theorem** **1.** *Let* α>0*,* k≥0 *and* t>0*. Then, the basis functions* $\phi_{k}\left(t,\alpha \right)$ *(8) are given by:*

(9) $$\phi_{k}\left(t,\alpha \right)=2e^{k}{\left(\frac{\sqrt{\alpha t}}{k}\right)}^{k}K_{k}\left(2\sqrt{\alpha t}\right)\;,$$

*where* $K_{k}\left(x\right)$ *is the modified Bessel function of the second kind [45,46] of integer order k.*

The first Function (9) is as follows

(10) $$\phi_{0}\left(t,\alpha \right)=2K_{0}\left(2\sqrt{\alpha t}\right)\;.$$

The modified Bessel functions of the second kind, and especially $K_{0}\left(x\right)$ [47], have many applications in science and engineering, for example, in physics to describe the flow of magneto-hydrodynamic (MHD) viscous fluid in a Darcy-type porous medium [48], in engineering to derive a closed analytical form of the model of a axial-flux permanent magnet machine with segmented multipole-Halbach PM array [49] and to describe the per-unit-length internal impedance of two-layer cylindrical conductors [50]. The applications of the Bessel functions in the description of the dynamic response of a mono-pile foundation in homogeneous soil and varied layered soil–rock conditions under horizontal dynamic loads [51], to obtain a fully coupled poroelastic solution for spherical indentation into a half space with an impermeable surface when the indenter is subjected to step displacement loading [52] and to express a distribution of the traveling distance in heterogeneous populations [53] come from material science and ecology. 

**Figure 1:**
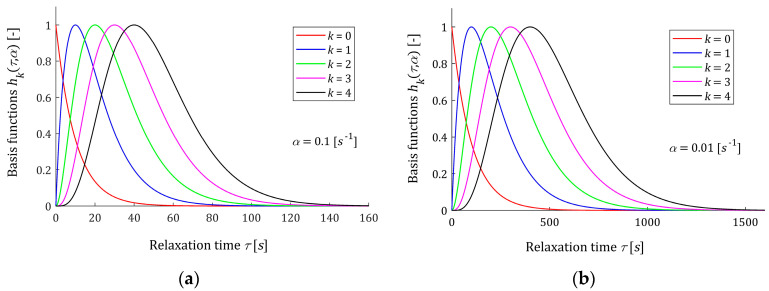
Basis functions $h_{k}\left(\tau ,\alpha \right)$ (3) and (4) of the relaxation spectrum model $H_{K}\left(\tau ,\alpha \right)$ (6) for time-scaling factors: (**a**) $\alpha =0.1\;\left[s^{-1}\right]$ and (**b**) $\alpha =0.01\;\left[s^{-1}\right]$, k=0,1,2,3,4.

**Figure 2:**
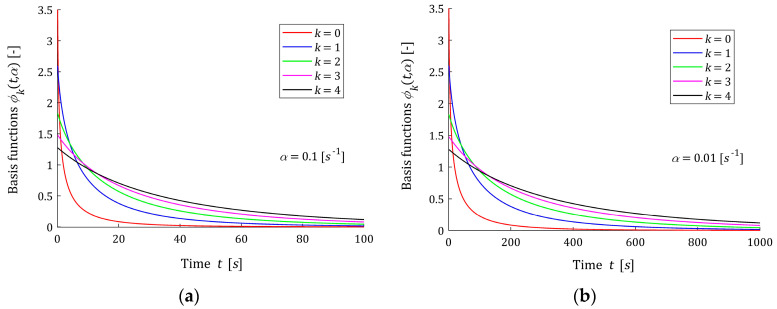
Basis functions $\phi_{k}\left(t,\alpha \right)$ (9) of the relaxation modulus model for time-scaling factors: (**a**) $\alpha =0.1\;\left[s^{-1}\right]$ and (**b**) $\alpha =0.01\;\left[s^{-1}\right]$, k=0,1,2,3,4.

**Figure 3:**
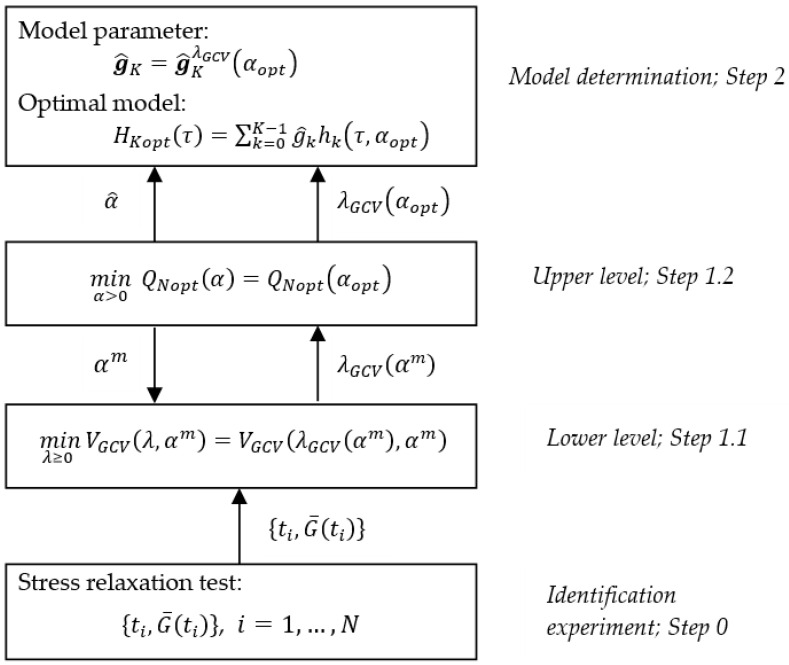
Hierarchical procedure of the two-level scheme for determination of the optimal spectrum relaxation model.

**Figure 4:**
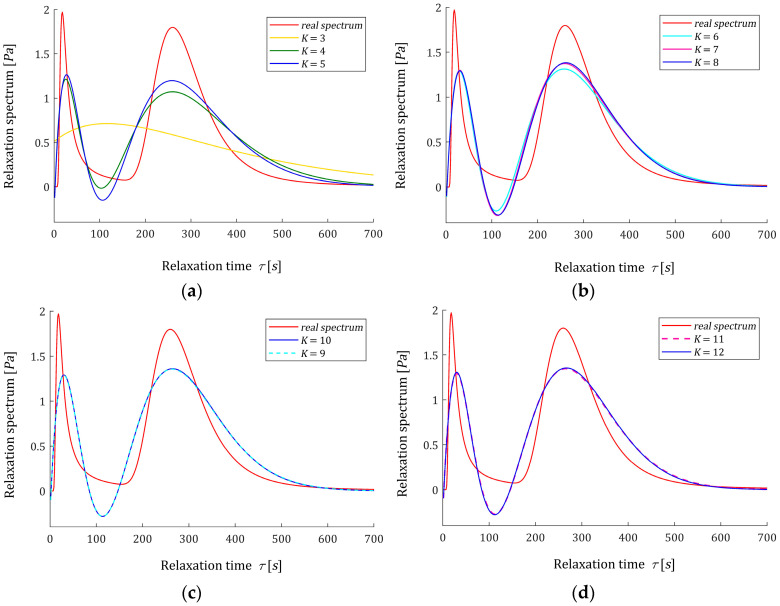
Relaxation time spectrum H(τ) (61) (solid red line) from the example and the corresponding models $H_{Kopt}\left(\tau \right)$ (46) for K summands of the model: (**a**) K=3,4,5; (**b**) K=6,7,8; (**c**) K=9,10; (**d**) K=11,12.

**Figure 5:**
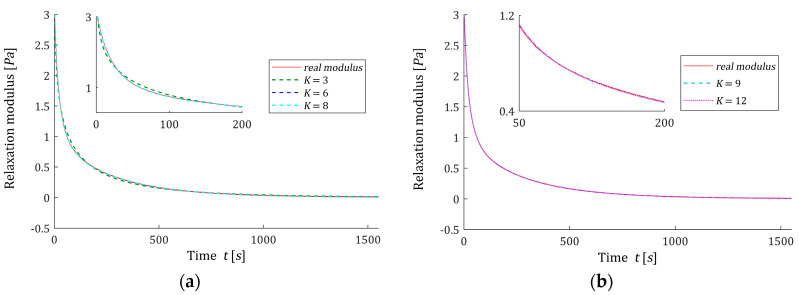
The measurements $\bar{G}\left(t_{i}\right)$ of the ‘real’ relaxation modulus G(t) (62) (red points) from the example and the optimal approximated models $G_{K}\left(t,\alpha_{opt}\right)$ (7) computed for ${\hat{g}}_{K}$ (45) for K summands of the model: (**a**) K=3,6,8; (**b**) K=9,12.

**Figure 6:**
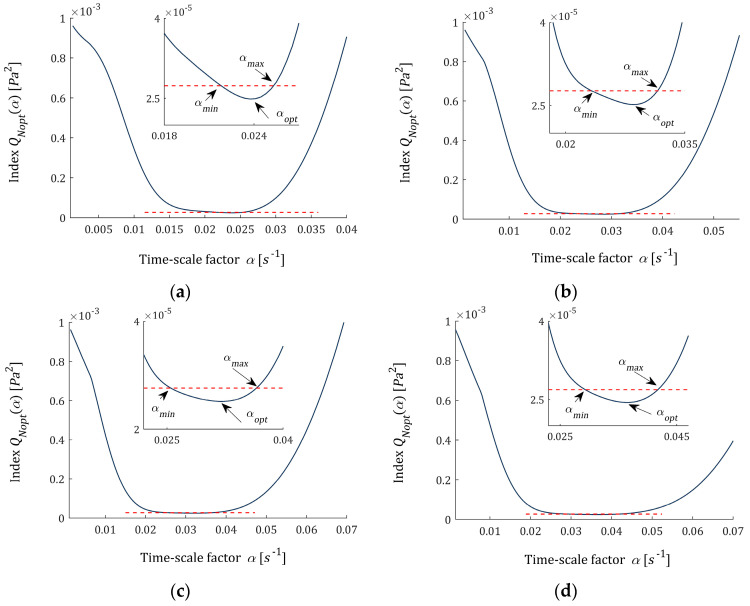
The identification index $Q_{Nopt}\left(\alpha \right)$ (35) (solid navy blue line) minimized in the upper level task (37) from the example for: (**a**) K=6, (**b**) K=8, (**c**) K=10, (**d**) K=12 summands of the relaxation spectrum model (6); on small subfigures, dashed red line of the value $\left(1+\varepsilon_{\alpha }\right)Q_{Nopt}\left(\alpha_{opt}\right)$ determines the interval $\left[\alpha_{min},\alpha_{max}\right]$ of suboptimal time-scale factors defined by (66); $\alpha_{opt}$ is the optimal time-scale factor solving problem (37) for the sub-optimality factor $\varepsilon_{\alpha }=10$%.

**Figure 7:**
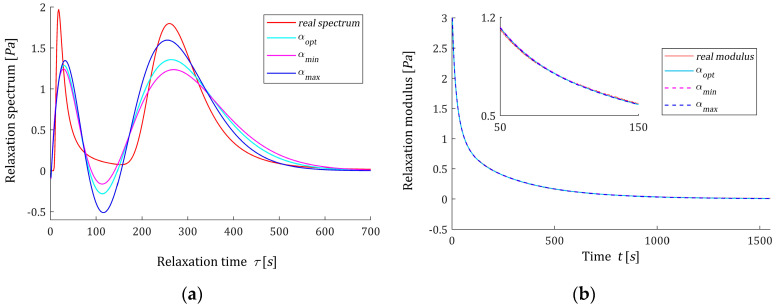
Relaxation time spectra and moduli from the example and the corresponding models for K=9 summands of the model: (**a**) ‘real’ spectrum H(τ) (61) (solid red line) and the models: optimal $H_{Kopt}\left(\tau \right)$ (46) and suboptimal $H_{K}\left(\tau ,\alpha_{min}\right)$ and $H_{K}\left(\tau ,\alpha_{max}\right)$; (**b**) ‘real’ modulus G(t) (62) (red points), the optimal approximated model $G_{K}\left(t,\alpha_{opt}\right)$ (7) and (45), the suboptimal models $G_{K}\left(t,\alpha_{min}\right)$ and $G_{K}\left(t,\alpha_{max}\right)$ computed for ${\hat{g}}_{K}^{\lambda_{GCV}}\left(\alpha_{min}\right)$ and ${\hat{g}}_{K}^{\lambda_{GCV}}\left(\alpha_{max}\right)$ according to (7).

Five first basis functions $h_{k}\left(\tau ,\alpha \right)$ (3) are shown in Figure 1 for two different values of the time-scaling factor α. Figure 2 shows the related functions $\phi_{k}\left(t,\alpha \right)$ (9). The basis functions $h_{k}\left(\tau ,\alpha \right)$ and $\phi_{k}\left(t,\alpha \right)$ are dimensionless. It is seen from Figure 1 that the maximum of each scaled basis function $h_{k}\left(\tau ,\alpha \right)$ (3) and (4) is equal one; however, the relaxation time $t^{max}$ corresponding to the maximum, for a given parameter α, depends on the index k according to the formula $t^{max}=k/\alpha$; i.e., grows with k. This means that increasing the number of model components K will allow for good modeling of multimodal spectra, which is confirmed by the example presented in the final part of the paper. Reducing the time-scale factor α shifts the spectrum maxima towards larger relaxation times. From Figure 2, it is seen that the Debye decay monotonicity of basis functions for the relaxation modulus model is in good agreement with the courses of the relaxation modulus obtained in an experiment for real materials; for example: concrete [10] (Figure 13), rubber [12] (Figure 2), elastic polyacrylamide hydrogels [28] (Figures 2a,b, 4a, A5, A7 and A8a), sugar beet [18] (Figure 1) and several foods [3] (Figures 3–39). 

#### 2.2.1. Positive Definiteness of the Basis Functions

The basis functions of the relaxation spectrum and modulus models are positive definite. For the functions $h_{k}\left(\tau ,\alpha \right)$ (3) and (4), these properties are obvious. Since, according to the property A.1 in [54], the Bessel functions of the second kind $K_{v}\left(x\right)$ are positive for x>0 and real v, the positive definiteness of the functions $\phi_{k}\left(t,\alpha \right)$ (9) and $\phi_{0}\left(t,\alpha \right)$ (10) directly result. 

#### 2.2.2. Asymptotic Properties of the Basis Functions

For x→0, the following asymptotic formula is found in the literature [45]:

(11) $$K_{k}\left(x\right)\sim \frac{1}{2}\Gamma \left(k\right){\left(\frac{x}{2}\right)}^{-k}\;,$$

for modified Bessel functions of the order k>0. Thus, for t→0, Formulas (11) and (9) imply

$$\phi_{k}\left(t,\alpha \right)\sim \Gamma \left(k\right){\left(\frac{e}{k}\right)}^{k}\;,$$

which means that for t near zero, the values of basis functions $\phi_{k}\left(t,\alpha \right)$ decrease with increasing index k; see Figure 2. 

For argument x→∞, the asymptotic exponential formula holds [54]

(12) $$K_{k}\left(x\right)\sim \sqrt{\frac{\pi }{2x}}e^{-x}\;.$$

From (9) and (12), the next asymptotic formula follows for large t and k≥1

(13) $$\phi_{k}\left(t,\alpha \right)\sim \frac{\sqrt{\pi }}{k^{k}}{\left(\sqrt{\alpha t}\right)}^{k-\frac{1}{4}}e^{-2\sqrt{\alpha t}+k}\;,$$

while for t→∞ and the first basis function, we have

(14) $$\phi_{0}\left(t,\alpha \right)\sim \sqrt{\frac{\pi }{\sqrt{\alpha t}}}e^{-2\sqrt{\alpha t}}\;\;.$$

The multiplication by power function ${\left(\sqrt{\alpha t}\right)}^{k-\frac{1}{4}}$ in (13) causes that the greater k is, the slower the basis function $\phi_{k}\left(t,\alpha \right)$ decreases, which is seen in Figure 2. The first basis function $\phi_{0}\left(t,\alpha \right)$ decreases faster than the exponential function, which is also confirmed by the analysis of the course of the basis functions in Figure 2.

By the asymptotic Formulas (13) and (14), the basis functions tend to 0 as t→∞; i.e., for an arbitrary, α>0 and k=0,1,2,…

(15) $$\underset{t\to \infty }{lim}\phi_{k}\left(t,\alpha \right)=0.$$

#### 2.2.3. Upper Bounds for the Basis Functions

In [54], Corollary 3.4, the following inequality is proved:

$$K_{0}\left(x\right)<\sqrt{\frac{\pi }{2x}}\;e^{-x},$$

whence

(16) $$\phi_{0}\left(t,\alpha \right)=2K_{0}\left(2\sqrt{\alpha t}\right)<\sqrt{\frac{\pi }{\sqrt{\alpha t}}}\;e^{-2\sqrt{\alpha t}}\;.$$

From Theorem 3.1 in [54], for k≥1 we have

$$K_{k}\left(x\right)<\frac{2^{k-1}\Gamma \left(k\right)}{x^{k+1}}.$$

This inequality applied into (9) gives the following upper bound

(17) $$\phi_{k}\left(t,\alpha \right)<2e^{k}{\left(\frac{\sqrt{\alpha t}}{k}\right)}^{k}\;\frac{2^{k-1}\Gamma \left(k\right)}{{\left(2\sqrt{\alpha t}\right)}^{k+1}}=\frac{e^{k}}{2k^{k}}\;\frac{\Gamma \left(k\right)}{\sqrt{\alpha t}}.$$

Note that having in mind the positive definiteness of the basis functions $\phi_{k}\left(t,\alpha \right)$, the limit (15) for the first function $\phi_{k}\left(t,\alpha \right)$ yields from (16), while, for any k≥1, can be derived from (17). 

#### 2.2.4. Monotonicity of the Basis Functions

As indicated above, the basis functions $h_{k}\left(\tau ,\alpha \right)$ (3) for k≥1 have a global maximum equal to 1 for the relaxation time $\tau =k\alpha^{-1}$, while the first function $h_{0}\left(\tau ,\alpha \right)$ (4) is monotonically decreasing. 

Since the basis function $\phi_{k}\left(t,\alpha \right)$ (9) is the product of a monotonically decreasing Bessel function $K_{k}\left(x\right)$ [54] of increasing argument $2\sqrt{\alpha t}$ and a monotonically increasing power function ${\left(\sqrt{\alpha t}\right)}^{k}$, its monotonicity is not obvious. By applying the differentiation formula, we obtain [54] (Equation (A.13)):

(18) $$\frac{d}{dx}\left[x^{v}K_{v}\left(x\right)\right]=-x^{v}K_{v-1}\left(x\right)$$

which holds for any real-valued order v, to (9), gives for integer k≥1

$$\frac{d}{dt}\left[\phi_{k}\left(t,\alpha \right)\right]=-4\alpha {\left(\frac{e}{2k}\right)}^{k}{\left(2\sqrt{\alpha t}\right)}^{k-1}K_{k-1}\left(2\sqrt{\alpha t}\right)\;,$$

whereas, having in mind the positive definiteness of the Bessel function $K_{k-1}\left(2\sqrt{\alpha t}\right)$ for all t>0, we immediately conclude that the function $\phi_{k}\left(t,\alpha \right)$ is monotonically decreasing; see Figure 2. For the first basis function $\phi_{0}\left(t,\alpha \right)$ (10), the next differentiation formula is [54] (Equation (A.14)):

(19) $$\frac{d}{dx}\left[K_{v}\left(x\right)\right]=-\frac{1}{2}\left[K_{v-1}\left(x\right)+K_{v+1}\left(x\right)\right],$$

which is satisfied for any real v, implies

$$\frac{d}{dt}\left[\phi_{0}\left(t,\alpha \right)\right]=-\sqrt{\frac{\alpha }{t}}\left[K_{-1}\left(2\sqrt{\alpha t}\right)+K_{1}\left(2\sqrt{\alpha t}\right)\right]\;,$$

which shows that $\phi_{0}\left(t,\alpha \right)$ is also a monotonically decreasing function. 

#### 2.2.5. Ranges of Applicability

In the models, the parameter α>0 is a time-scaling factor. The following rule holds: the lower the parameter α, the greater the relaxation times. The above is illustrated by Figure 1 and Figure 2. Through the optimal choice of the scaling factor, the best fit of the model to the experimental data is achieved, which will be the subject of study in Section 3.2. 

Following [40], on the basis of the relaxation modulus course, the range of applicability is specified as the time t, for which the first K basis functions $\phi_{k}\left(t,\alpha \right)$ no longer permanently exceed; i.e., for any θ>t, ε=0.5% of its maximum value. Specifically,

(20) $$t_{app}\left(\alpha \right)=\underset{0\leq k\leq K-1}{max}\underset{t>0}{min}\;\left\{t:\left|\phi_{k}\left(\theta ,\alpha \right)\right|\leq 0.005\cdot \phi_{kmax}\left(\alpha \right)\;\mathrm{for}\;\mathrm{any}\;\theta \geq \;t\right\},$$

where

$$\phi_{kmax}\left(\alpha \right)=\underset{t\geq 0}{max}\;\left|\phi_{k}\left(t,\alpha \right)\right|.$$

Similarly, in [40], the range of applicability specified directly for the relaxation times τ was defined on the basis of the variability in the basis functions $h_{k}\left(\tau \right)$; i.e.,

(21) $$\tau_{app}\left(\alpha \right)=\underset{0\leq k\leq K-1}{max}\underset{\tau >0}{min}\;\left\{\tau :\left|h_{k}\left(\vartheta ,\alpha \right)\right|\leq 0.005\cdot h_{kmax}\left(\alpha \right)\;\mathrm{for}\;\mathrm{any}\;\vartheta \geq \;\tau \right\},$$

with $h_{kmax}\left(\alpha \right)$ defined by

$$h_{kmax}\left(\alpha \right)=\underset{\tau \geq 0}{max}\;\left|h_{k}\left(\tau ,\alpha \right)\right|.$$

The times $t_{app}\left(\alpha \right)$ (20) and $\tau_{app}\left(\alpha \right)$ (21) for different values of α are summarized in Table 1 for K=5 and K=12. The same data for K=6,…,11 are given in Table A1 in Appendix B.

A review of the data from these tables shows that for any fixed α, both $\tau_{app}\left(\alpha \right)$ and $t_{app}\left(\alpha \right)$ grow almost linearly with the number of model summands K.

### 2.3. Least-Squares Regularized Identification 

Identification consists of the selection, within the chosen class of models given by (6) and (7), of such a model that ensures the best fit to the measurement results. Suppose a certain identification experiment (stress relaxation test [2,3]) resulted in a set of measurements of the relaxation modulus $\left\{\bar{G}\left(t_{i}\right)=G\left(t_{i}\right)+z\left(t_{i}\right)\right\}$ at the sampling instants $t_{i}\geq 0$, i=1,…,N, where $z\left(t_{i}\right)$ is the measurement noise. It is assumed that the number of measurements N≥K. As a measure of the model (7) accuracy, the quadratic index is taken

(22) $$Q_{N}\left(g_{K},\alpha \right)=\sum_{i=1}^{N}{\left[\bar{G}\left(t_{i}\right)-G_{K}\left(t_{i},\alpha \right)\right]}^{2},$$

where $g_{K}={\left[\begin{array}{ccc} g_{0} & \cdots & g_{K-1} \end{array}\right]}^{T}$ is an K-element vector of unknown coefficients of the models (6) and (7). The identification index (22) is rewritten in the compact form as

(23) $$Q_{N}\left(g_{K},\alpha \right)={\left\|{\bar{G}}_{N}-\Phi_{N,K}\left(\alpha \right)g_{K}\right\|}_{2}^{2},$$

where

(24) $$\Phi_{N,K}\left(\alpha \right)=\left[\begin{array}{ccc} \phi_{0}\left(t_{1},\alpha \right) & \cdots & \phi_{K-1}\left(t_{1},\alpha \right)\\ \vdots & \ddots & \vdots \\ \phi_{0}\left(t_{N},\alpha \right) & \cdots & \phi_{K-1}\left(t_{N},\alpha \right) \end{array}\right],\;{\bar{G}}_{N}=\left[\begin{array}{c} \bar{G}\left(t_{1}\right)\\ \vdots \\ \bar{G}\left(t_{N}\right) \end{array}\right]$$

and ${\left\|\cdot \right\|}_{2}$ denotes the square norm in the real Euclidean space ${ℛ}^{N}$. Thus, the optimal identification of the relaxation spectrum in the class of models defined by (6) and (7) consists of determining the model parameter $g_{K}$ through solving the following optimization task:

(25) $$\underset{g_{K}\in {ℛ}^{K}}{min}\;\;\;\;\;{\left\|{\bar{G}}_{N}-\Phi_{N,K}\left(\alpha \right)\;g_{K}\right\|}_{2}^{2}.$$

The matrix $\Phi_{N,K}\left(\alpha \right)$ is usually ill-conditioned. In consequence, the problem (25) is ill posed in the sense of Hadamard [42]. The parameter $g_{K}$ minimizing identification index (23) is not unique, and even the normal (with the lowest Euclidean norm) solution of (25) is a non-continuous and unbounded function of the measurement vector ${\bar{G}}_{N}$. Therefore, when the data are noisy, even small changes in ${\bar{G}}_{N}$ would lead to arbitrarily large artefacts in optimal model parameters $g_{K}$. To deal with the ill-posedness, Tikhonov regularization [55], replacing the original ill-posed problem (25) by a nearby problem with a modified square functional of the form:

(26) $$\underset{g_{K}\in {ℛ}^{K}}{min}{\left\|{\bar{G}}_{N}-\Phi_{N,K}\left(\alpha \right)\;g_{K}\right\|}_{2}^{2}+\lambda {\left\|g_{K}\right\|}_{2}^{2},$$

where λ>0 is a regularization parameter, can be used. The regularized task (26) is well-posed; that is, the solution always exists, is unique and continuously depends on both the matrix $\Phi_{N,K}$ and on the measurement data ${\bar{G}}_{N}$. The parameter vector solving (26) is given by:

(27) $${\bar{g}}_{K}^{\lambda }\left(\alpha \right)={\left(\Phi_{N,K}^{T}\left(\alpha \right)\Phi_{N,K}\left(\alpha \right)+\lambda I_{K,K}\right)}^{-1}\Phi_{N,K}^{T}\left(\alpha \right){\bar{G}}_{N},$$

where $I_{K,K}$ is the K-dimensional identity matrix. 

Following [40], the generalized cross-validation GCV [42,56] is applied, according to which, the best regularization parameter is [42,56]:

(28) $$\lambda_{GCV}\left(\alpha \right)=min\left\{\lambda :\;\lambda =arg\;\underset{\lambda \geq 0}{min}\;V_{GCV}\left(\lambda ,\alpha \right)\right\},$$

where the GCV functional is defined by [56]

(29) $$V_{GCV}\left(\lambda ,\alpha \right)=\frac{{\left\|\Xi \left(\lambda ,\alpha \right){\bar{G}}_{N}\right\|}_{2}^{2}}{tr{\left[\Xi \left(\lambda ,\alpha \right)\right]}^{2}},$$

with the matrix

(30) $$\Xi \left(\lambda ,\alpha \right)=I_{N,N}-\Phi_{N,K}\left(\alpha \right){\left(\Phi_{N,K}^{T}\left(\alpha \right)\Phi_{N,K}\left(\alpha \right)+\lambda I_{K,K}\right)}^{-1}\Phi_{N,K}^{T}\left(\alpha \right).$$

Here, $\Xi \left(\lambda ,\alpha \right){\bar{G}}_{N}$ is the residual vector for the regularized solution (27); tr[Ξ(λ,α)] denotes the trace of the symmetric matrix Ξ(λ,α). The optimization problem in (28) has a unique solution, and the resulting parameter ${\bar{g}}_{K}^{\lambda_{GCV}\left(\alpha \right)}\left(\alpha \right)$ differs the least from the normal solution of the problem (26) that we would obtain for the ideal (not noise-corrupted) measurements of the relaxation modulus [56].

## 3. Results and Discussion

In this section, the necessary optimality condition for the regularized identification task with GCV’s choice of regularization parameter is given in the form of an algebraic nonlinear equation. Next, the problem of the choice of the optimal scale factor is stated, and the respective necessary optimality condition is derived. A hierarchical two-level identification scheme with the optimal choice of the scale factor is proposed. The numerical realization and the application of a singular value decomposition technique are discussed. A complete computational procedure is outlined. The analysis of the smoothing of the model and model accuracy for noisy measurements of the relaxation modulus is presented. 

### 3.1. Necessary Optimality Condition 

The GCV functional (29) is a differentiable function of the regularization parameter λ. The necessary optimality condition for the minimization task solved in (28) is derived in Appendix A.3.

**Theorem** **2.** *The optimal regularization parameter* $\lambda_{GCV}\left(\alpha \right)$ *solves the following equation:*

(31) $${\bar{G}}_{N}^{T}{\left(\Psi \left(\alpha \right)+\lambda I_{N,N}\right)}^{-3}{\bar{G}}_{N}tr\left[{\left(\Psi \left(\alpha \right)+\lambda I_{N,N}\right)}^{-1}\right]={\bar{G}}_{N}^{T}(\Psi \left(\alpha \right)+\;\lambda I_{N,N}){}^{-2}{\bar{G}}_{N}\;tr\left[{\left(\Psi \left(\alpha \right)+\lambda I_{N,N}\right)}^{-2}\right].$$

*where*

(32) $$\Psi \left(\alpha \right)=\Phi_{N,K}\left(\alpha \right)\Phi_{N,K}^{T}\left(\alpha \right).$$

In Appendix A.4, the following formula:

(33) $$\frac{\partial V_{GCV}\left(\lambda ,\alpha \right)}{\partial \lambda }=\;\frac{-2{\bar{G}}_{N}^{T}{\left(\Psi \left(\alpha \right)+\lambda I_{N,N}\right)}^{-3}\;{\bar{G}}_{N}tr\left[{\left(\Psi \left(\alpha \right)+\lambda I_{N,N}\right)}^{-1}\right]+2{\bar{G}}_{N}^{T}{\left(\Psi \left(\alpha \right)+\lambda I_{N,N}\right)}^{-2}{\bar{G}}_{N}tr\left[{\left(\Psi \left(\alpha \right)+\lambda I_{N,N}\right)}^{-2}\right]}{{\left[tr\left[{\left(\Psi \left(\alpha \right)+\lambda I_{N,N}\right)}^{-1}\right]\right]}^{3}}$$

is developed, describing the derivative of the GCV functional (29) minimized in (28) directly as a function of matrices ${\bar{G}}_{N}$ and Ψ(α). 

### 3.2. Choice of the Time-Scale Factor

The optimal regularization parameter $\lambda_{GCV}\left(\alpha \right)$ and matrix $\Phi_{N,K}\left(\alpha \right)$ depend on the time-scale factor. Therefore, the optimal model parameter

(34) $${\hat{g}}_{K}^{\lambda_{GCV}}\left(\alpha \right)={{\bar{g}}_{K}^{\lambda }\left(\alpha \right)|}_{\lambda =\lambda_{GCV}\left(\alpha \right)}$$

and the optimal identification index

(35) $$Q_{Nopt}\left(\alpha \right)=Q_{N}\left({\hat{g}}_{K}^{\lambda_{GCV}}\left(\alpha \right),\alpha \right)={\left\|{\bar{G}}_{N}-\Phi_{N,K}\left(\alpha \right){\hat{g}}_{K}^{\lambda_{GCV}}\left(\alpha \right)\right\|}_{2}^{2}$$

also depend on α. By the optimal choice of the scaling factor, the best fit of the model to the experimental data can be achieved. By (27), (30) and (34), and having (A9) in mind, the index $Q_{Nopt}\left(\alpha \right)$ (35) is expressed by the following formula:

(36) $$Q_{Nopt}\left(\alpha \right)=\lambda_{GCV}^{2}\left(\alpha \right){\bar{G}}_{N}^{T}{\left(\Psi \left(\alpha \right)+\lambda_{GCV}\left(\alpha \right)I_{N,N}\right)}^{-2}{\bar{G}}_{N}.$$

The smaller the index $Q_{Nopt}\left(\alpha \right)$, the smaller the model error results. Thus, the problem of the choice of the best time-scale factor $\alpha_{opt}$ takes the form

(37) $$\underset{\alpha >0}{min}\;Q_{Nopt}\left(\alpha \right)=Q_{Nopt}\left(\alpha_{opt}\right).$$

Before we state the necessary optimality condition of the task (37), we prove the following result concerning the basis functions $\phi_{k}\left(t,\alpha \right)$ as the functions of the time-scale factor. The proof is given in Appendix A.5.

**Theorem** **3.** *Let* α>0 *and* t>0*. Then, for* k≥1*, derivatives of the basis functions* $\phi_{k}\left(t,\alpha \right)$ *(9) with respect to the parameter* α *are given by:*

(38) $$\frac{d}{d\alpha }\left[\phi_{k}\left(t,\alpha \right)\right]=-e\left(\frac{t}{k}\right){\left(\frac{k-1}{k}\right)}^{k-1}\phi_{k-1}\left(t,\alpha \right).$$

*For* k=0, *the following formula holds:*

(39) $$\frac{d}{d\alpha }\left[\phi_{0}\left(t,\alpha \right)\right]=-\frac{1}{e\alpha }\phi_{1}\left(t,\alpha \right)\;.$$

Thus, positive definite functions $\phi_{k}\left(t,\alpha \right)$ are monotonically decreasing functions of α for any fixed t>0. 

The following necessary optimality condition is derived in Appendix A.6:

**Theorem** **4.** *The optimal time-scale factor* $\alpha_{opt}$ *satisfies the following equation:*

(40) $$\lambda_{GCV}\left(\alpha \right){\bar{G}}_{N}^{T}\Upsilon {\left(\alpha \right)}^{-1}\Omega_{N,K}\left(\alpha \right)\Upsilon {\left(\alpha \right)}^{-2}{\bar{G}}_{N}=\frac{d\lambda_{GCV}\left(\alpha \right)}{d\alpha }{\bar{G}}_{N}^{T}\Upsilon {\left(\alpha \right)}^{-2}{\bar{G}}_{N}-\;\lambda_{GCV}\left(\alpha \right)\frac{d\lambda_{GCV}\left(\alpha \right)}{d\alpha }{\bar{G}}_{N}^{T}\Upsilon {\left(\alpha \right)}^{-3}{\bar{G}}_{N},$$

*where derivative* $\frac{d\lambda_{GCV}\left(\alpha \right)}{d\alpha }$ *is given by the equation*

(41) $$\frac{d\lambda_{GCV}\left(\alpha \right)}{d\alpha }\{3{\bar{G}}_{N}^{T}\Upsilon {\left(\alpha \right)}^{-4}{\bar{G}}_{N}tr\left[\Upsilon {\left(\alpha \right)}^{-1}\right]-{\bar{G}}_{N}^{T}\Upsilon {\left(\alpha \right)}^{-3}{\bar{G}}_{N}tr\left[\Upsilon {\left(\alpha \right)}^{-2}\right]\;-2{\bar{G}}_{N}^{T}\Upsilon {\left(\alpha \right)}^{-2}{\bar{G}}_{N}\;tr\left[\Upsilon {\left(\alpha \right)}^{-3}\right]\}=\;2{\bar{G}}_{N}^{T}\Upsilon {\left(\alpha \right)}^{-2}\Omega_{N,K}\left(\alpha \right)\Upsilon {\left(\alpha \right)}^{-1}{\bar{G}}_{N}\;tr\left[\Upsilon {\left(\alpha \right)}^{-2}\right]+\;2{\bar{G}}_{N}^{T}\Upsilon {\left(\alpha \right)}^{-2}{\bar{G}}_{N}\;tr[\Omega_{N,K}\left(\alpha \right)\Upsilon {\left(\alpha \right)}^{-3}]-\;2{\bar{G}}_{N}^{T}\Upsilon {\left(\alpha \right)}^{-3}\Omega_{N,K}\left(\alpha \right)\Upsilon {\left(\alpha \right)}^{-1}{\bar{G}}_{N}tr\left[\Upsilon {\left(\alpha \right)}^{-1}\right]-\;{\bar{G}}_{N}^{T}\Upsilon {\left(\alpha \right)}^{-2}\Omega_{N,K}\left(\alpha \right)\Upsilon {\left(\alpha \right)}^{-2}{\bar{G}}_{N}tr\left[\Upsilon {\left(\alpha \right)}^{-1}\right]-{\bar{G}}_{N}^{T}\Upsilon {\left(\alpha \right)}^{-3}{\bar{G}}_{N}tr\left[\Omega_{N,K}\left(\alpha \right)\Upsilon {\left(\alpha \right)}^{-2}\right],$$

*with symmetric matrices*

(42) $$\Upsilon \left(\alpha \right)=\Psi \left(\alpha \right)+\lambda_{GCV}\left(\alpha \right)I_{N,N},$$

(43) $$\Omega_{N,K}\left(\alpha \right)=\frac{d}{d\alpha }\Psi \left(\alpha \right)=\Theta_{N,K}\left(\alpha \right)\Phi_{N,K}^{T}\left(\alpha \right)+\Phi_{N,K}\left(\alpha \right)\Theta_{N,K}^{T}\left(\alpha \right).$$

*where* Ψ(α) *is defined by (32) and*

(44) $$\Theta_{N,K}\left(\alpha \right)=\frac{d}{d\alpha }\Phi_{N,K}\left(\alpha \right)=\;-\left[\begin{array}{cccc} \frac{1}{e\alpha }\phi_{1}\left(t_{1},\alpha \right) & et_{1}\phi_{0}\left(t_{1},\alpha \right) & \cdots & e\left(\frac{t_{1}}{K-1}\right){\left(\frac{K-2}{K-1}\right)}^{K-2}\phi_{K-2}\left(t_{1},\alpha \right)\\ \vdots & \vdots & \ddots & \vdots \\ \frac{1}{e\alpha }\phi_{1}\left(t_{N},\alpha \right) & et_{N}\phi_{0}\left(t_{N},\alpha \right) & \cdots & e\left(\frac{t_{N}}{K-1}\right){\left(\frac{K-2}{K-1}\right)}^{K-2}\phi_{K-2}\left(t_{N},\alpha \right) \end{array}\right].$$

### 3.3. Two-Level Identification Scheme

To find the optimal time-scale factor $\alpha_{opt}$ and the optimal model of the relaxation time spectrum, the following two-level scheme is applied: 

#### 3.3.1. Lower Level

Given time-scale factor α>0, find regularization parameter $\lambda_{GCV}\left(\alpha \right)$ solving the GCV minimization task (28). 

#### 3.3.2. Upper Level

Find the time-scale factor $\alpha_{opt}>0$ minimizing identification index $Q_{Nopt}\left(\alpha \right)$ (36), i.e., solving optimization task (37). Take

(45) $${\hat{g}}_{K}={\hat{g}}_{K}^{\lambda_{GCV}}\left(\alpha_{opt}\right),$$

where ${\hat{g}}_{K}^{\lambda_{GCV}}\left(\alpha_{opt}\right)$ is defined by (34) as a parameter of the best model of the relaxation time spectrum. 

Having the optimal model parameter ${\hat{g}}_{K}$, the optimal model of the relaxation spectrum is determined according to the formula resulting directly from (6):

(46) $$H_{Kopt}\left(\tau \right)=H_{K}\left(\tau ,\alpha_{opt}\right)=\sum_{k=0}^{K-1}{\hat{g}}_{k}h_{k}\left(\tau ,\alpha_{opt}\right),$$

where ${\hat{g}}_{k}$ are elements of the vector ${\hat{g}}_{K}$. 

To design numerical realization of the scheme, we need:

1. A numerical procedure for solving the lower-level GCV minimization task (28);
2. An iterative scheme for solving the upper-level problem (37) of choosing the best time-scale factor.

For any given parameter α, the GCV function $V_{GCV}\left(\lambda ,\alpha \right)$ (29) is differentiable with respect to λ. Partial derivative $\frac{\partial V_{GCV}\left(\lambda ,\alpha \right)}{\partial \lambda }$ is given by (33) as a function of the experiment data ${\bar{G}}_{N}$ and the matrix Ψ(α) (32), which depends on the time instants $t_{i}$ used in the relaxation experiment and on the time-scaling factor. An arbitrary gradient optimization method can be implemented to solve the GCV minimization task (28). Additionally, an arbitrary gradient method can be used to solve optimization problem (37); the derivative of the index $Q_{Nopt}\left(\alpha \right)$ (36) is described by Formula (A24) derived in Appendix A.6. However, the optimal parameter $\alpha_{opt}$ can be also found by solving the necessary optimality condition from Theorem 4, i.e., the two scalar algebraic Equations (40) and (41), in fact.

### 3.4. Algebraic Background of the Identification Scheme

Formulas (27)–(30), fundamental for lower-level optimization task (28), are elegant but generally unsuitable for computational purposes. Following [40], the singular value decomposition (SVD) technique [57] will be applied. Let SVD of the N×K dimensional matrix $\Phi_{N,K}\left(\alpha \right)$ take the form [57]:

(47) $$\Phi_{N,K}\left(\alpha \right)=U\left(\alpha \right)\Sigma \left(\alpha \right)\;V{\left(\alpha \right)}^{T},$$

where $\Sigma \left(\alpha \right)=diag\left(\sigma_{1}\left(\alpha \right),\ldots ,\sigma_{r\left(\alpha \right)}\left(\alpha \right),0,\ldots ,0\right)\epsilon {ℛ}^{N,K}$ is a diagonal matrix containing the non-zero singular values $\sigma_{1}\left(\alpha \right),\ldots ,\sigma_{r\left(\alpha \right)}\left(\alpha \right)$ of the matrix $\Phi_{N,K}\left(\alpha \right)$, matrices $V\left(\alpha \right)\in {ℛ}^{K,K}$ and $U\left(\alpha \right)\in {ℛ}^{N,N}$ are orthogonal and $r\left(\alpha \right)=rank\left[\Phi_{N,K}\left(\alpha \right)\right]<N$. Taking advantage of the diagonal structure of Σ(α) and orthogonality of the matrices V(α) and U(α), it may be simply proved that the parameter ${\bar{g}}_{K}^{\lambda }\left(\alpha \right)$ (27) is given by

(48) $${\bar{g}}_{K}^{\lambda }\left(\alpha \right)=V\left(\alpha \right)\Lambda_{\lambda }\left(\alpha \right)\;U{\left(\alpha \right)}^{T}\;{\bar{G}}_{N},$$

where K×N diagonal matrix $\Lambda_{\lambda }\left(\alpha \right)$ is as follows:

(49) $$\Lambda_{\lambda }\left(\alpha \right)=diag\left(\frac{\sigma_{1}\left(\alpha \right)}{{\left[\sigma_{1}\left(\alpha \right)\right]}^{2}+\lambda },\ldots ,\frac{\sigma_{r\left(\alpha \right)}\left(\alpha \right)}{{\left[\sigma_{r\left(\alpha \right)}\left(\alpha \right)\right]}^{2}+\lambda },0,\ldots ,0\right).$$

Using SVD (47) and introducing N dimensional vector $Y\left(\alpha \right)=U{\left(\alpha \right)}^{T}\;{\bar{G}}_{N}$, the GCV function (29) is also expressed by a convenient analytical formula:

(50) $$V_{GCV}\left(\lambda ,\alpha \right)=\frac{\left[\sum_{i=1}^{r\left(\alpha \right)}\frac{\lambda^{2}{\left[y_{i}\left(\alpha \right)\right]}^{2}}{{\left({\left[\sigma_{i}\left(\alpha \right)\right]}^{2}+\lambda \right)}^{2}}+\sum_{i=r\left(\alpha \right)+1}^{N}\;{\left[y_{i}\left(\alpha \right)\right]}^{2}\right]}{{\left[N-r\left(\alpha \right)+\sum_{i=1}^{r\left(\alpha \right)}\frac{\lambda }{\left({\left[\sigma_{i}\left(\alpha \right)\right]}^{2}+\lambda \right)}\right]}^{2}},$$

as a function of the singular values $\sigma_{i}\left(\alpha \right)$ and elements $y_{i}\left(\alpha \right)$ of the vector Y(α). 

Similarly, the identification index $Q_{Nopt}\left(\alpha \right)$ (36) minimized in the upper level of the scheme is expressed using the SVD (47). Since, by virtue of (47) and (32) we have

$${\left(\Psi \left(\alpha \right)+\lambda_{GCV}\left(\alpha \right)I_{N,N}\right)}^{-2}=U\left(\alpha \right){\left[\Sigma \left(\alpha \right)\;\Sigma {\left(\alpha \right)}^{T}+\lambda_{GCV}\left(\alpha \right)I_{N,N}\right]}^{-2}U{\left(\alpha \right)}^{T},$$

the index ${\hat{Q}}_{N}\left(\alpha \right)$ is expressed as

(51) $$Q_{Nopt}\left(\alpha \right)=\lambda_{GCV}^{2}\left(\alpha \right)\left[\sum_{i=1}^{r\left(\alpha \right)}\frac{{\left[y_{i}\left(\alpha \right)\right]}^{2}}{{\left({\left[\sigma_{i}\left(\alpha \right)\right]}^{2}+\lambda_{GCV}\left(\alpha \right)\right)}^{2}}+\sum_{i=r\left(\alpha \right)+1}^{N}\;{\left[y_{i}\left(\alpha \right)\right]}^{2}\right].$$

### 3.5. Computational Algorithm for Model Identification

To design a numerical algorithm of the scheme, the communication between the levels should also be resolved. The computations must be arranged hierarchically in a two-level structure, i.e., for each iteration of the minimization procedure at the upper level, the whole numerical procedure must be realized for the lower-level GCV task (28). The complete computational procedure for determining the optimal model is given below.

**Step 0:** Perform the experiment—stress relaxation test [1,2,3,28]—and record the measurements $\bar{G}\left(t_{i}\right)$, i=1,…,N, of the relaxation modulus at times $t_{i}\geq 0$.
**Step 1:** Determine the optimal regularization parameter $\lambda_{GCV}\left(\alpha_{opt}\right)$ in the following two-level computations.
  **Step 1.0**: Choose the initial point $\alpha^{0}$ for the numerical procedure applied to solve the upper-level task (37).
  **Step 1.1**: Let $\alpha^{m}$ be the m-th iterate in the numerical procedure chosen to solve the upper-level task (37). For $\alpha =\alpha^{m}$, solve the lower-level minimization task (28) according to the chosen numerical optimization procedure and determine the regularization parameter $\lambda_{GCV}\left(\alpha^{m}\right)$. The algebraic formula $V_{GCV}\left(\lambda ,\alpha \right)$ (50) is applied.
  **Step 1.2**: Using $\lambda_{GCV}\left(\alpha^{m}\right)$, compute, according to the numerical procedure selected to solve the upper-level task (37), with the index $Q_{Nopt}\left(\alpha \right)$ described by (51), the new parameter $\alpha^{m+1}$, which is the next approximation of $\alpha_{opt}$. If for $\alpha^{m+1}$ the stopping rule of the chosen numerical procedure is satisfied, i.e.,
    $$\left|\alpha^{m+1}-\alpha^{m}\right|\leq \varepsilon_{1}$$
    or
    $$\left|Q_{Nopt}\left(\alpha^{m+1}\right)-Q_{Nopt}\left(\alpha^{m}\right)\right|\leq \varepsilon_{2},$$
    where $\varepsilon_{1}$ and $\varepsilon_{2}$ are preselected small positives, put $\alpha_{opt}=\alpha^{m}$ as the optimal time-scale factor, $\lambda_{GCV}\left(\alpha^{m}\right)$ as $\lambda_{GCV}\left(\alpha_{opt}\right)$, and go to Step 2. Otherwise, return to Step 1.1 and continue the computations for $\alpha =\alpha^{m+1}$.
**Step 2:** Compute the vector of the optimal model parameters ${\hat{g}}_{K}$ according to (45) and the best model of the relaxation spectrum $H_{Kopt}\left(\tau \right)$ given by (46).

**Remark** **1.** *The appealing feature of the scheme is that only the values of* $\lambda_{GCV}\left(\alpha^{m}\right)$*, not the related parameters vector* ${\hat{g}}_{K}^{\lambda_{GCV}}\left(\alpha^{m}\right)$ *(34), are used for* $\alpha^{m}$ *in successive iterations of the numerical procedure solving the upper-level task (37).*

**Remark** **2.** *The regularization parameter* $\lambda_{GCV}\left(\alpha^{m}\right)$ *resulting from the lower-level minimization task (28) in each iteration of the upper level is the solution of the GCV problem (28) for current* $\alpha^{m}$*. Thus, the respective vector* ${\hat{g}}_{K}^{\lambda_{GCV}}\left(\alpha^{m}\right)$ *(34) can be treated as an approximate solution of the overall identification problem*.

**Remark** **3.** *The selection of the initial time-scaling factor* $\alpha^{0}$ *in Step 1.0 may be based on the data concerning model applicability summarized in Table 1 and Table A1 or* $\alpha^{0}$ *can be selected by comparison, for different values of* α*, the first basis function* $\phi_{k}\left(t,\alpha \right)$ *(9), with the experiment results* $\left\{\bar{G}\left(t_{i}\right)\right\}$.

**Remark** **4.** *The SVD (47) of the matrix* $\Phi_{N,K}\left(\alpha \right)$*, of computational complexity* $O\left(NK^{2}\right)$ *[57], must be computed only once for successive* $\alpha^{m}$ *generated in Step 1.1. SVD is accessible in the form of optimized numerical procedures in most commonly used computational packets.*

The above procedure and communication between the levels are illustrated in Figure 3.

### 3.6. Analysis

Recently, a class of robust algorithms of the approximation of the continuous spectrum of relaxation frequencies by finite series of orthonormal functions has been developed and analyzed in detail in [40]. However, these results were derived using the orthogonality of the basis functions, so they are not applicable here. Therefore, both the analysis of the smoothing of the model and model accuracy for noisy measurements of the relaxation modulus must be carried out anew here.

#### 3.6.1. Smoothness

The purpose of regularization relies on the stabilization of the resulting vector ${\bar{g}}_{K}^{\lambda }\left(\alpha \right)$ (27). Since the basis functions $h_{k}\left(\tau ,\alpha \right)$ (3) and (4) are such that $h_{k}\left(\tau ,\alpha \right)\leq 1$ for any arguments, the following inequality

$$\underset{\tau \geq 0}{max}\left|H_{K}\left(\tau ,\alpha \right)\right|\leq \sum_{k=0}^{K-1}\left|g_{k}\right|$$

holds for an arbitrary time-scale factor, which means that the smoothing of the vector of model parameters results in the limitation of the respective relaxation spectrum. The mechanism of the vector ${\bar{g}}_{K}^{\lambda }\left(\alpha \right)$ (27) stabilization via Tikhonov regularization is explained in many papers, for example, [20,40,55]. The following rule holds: the greater the regularization parameter λ is, the more highly bounded the fluctuations of the vector ${\bar{g}}_{K}^{\lambda }\left(\alpha \right)$ are; see [40].

The norm ${\left\|H_{K}\left(\tau ,\alpha \right)\right\|}_{2}$ is a measure of smoothing of the relaxation spectrum model, where ${\left\|\cdot \right\|}_{2}$ also means the square norm in $L^{2}(0,\infty )$. In Appendix A.7, the following proposition is proved:

**Proposition** **1.** *For an arbitrary time-scale factor* α *and arbitrary vector of model parameters* $g_{K}$*, we have*

(52) $${\left\|H_{K}\left(\tau ,\alpha \right)\right\|}_{2}^{2}=g_{K}^{T}\Gamma \left(\alpha \right)g_{K}=\frac{1}{2\alpha }g_{K}^{T}\Gamma_{1}g_{K},$$

*where* K×K *symmetric real matrices,* Γ(α) *described by (A39) and* $\Gamma_{1}$ *given by*

(53) $$\Gamma_{1}=\left[\begin{array}{cccccc} 1 & \frac{e}{2} & \cdots & {\left(\frac{e}{2}\right)}^{j}\frac{j!}{j^{j}} & \cdots & {\left(\frac{e}{2}\right)}^{K-1}\frac{\left(K-1\right)!}{{\left(K-1\right)}^{K-1}}\\ \frac{e}{2} & 2{\left(\frac{e}{2}\right)}^{2} & \cdots & {\left(\frac{e}{2}\right)}^{1+j}\frac{\left(1+j\right)!}{\;j^{j}} & \cdots & {\left(\frac{e}{2}\right)}^{K}\frac{\left(K\right)!}{\;{\left(K-1\right)}^{K-1}}\\ \vdots & \vdots & \ddots & \vdots & \ddots & \vdots \\ {\left(\frac{e}{2}\right)}^{k}\frac{k!}{k^{k}} & {\left(\frac{e}{2}\right)}^{k+1}\frac{\left(k+1\right)!}{k^{k}} & \cdots & {\left(\frac{e}{2}\right)}^{k+j}\frac{\left(k+j\right)!}{k^{k}\;j^{j}} & \cdots & {\left(\frac{e}{2}\right)}^{k+K-1}\frac{\left(k+K-1\right)!}{k^{k}\;{\left(K-1\right)}^{K-1}}\\ \vdots & \vdots & \vdots & \vdots & \ddots & \vdots \\ {\left(\frac{e}{2}\right)}^{K-1}\frac{\left(K-1\right)!}{{\left(K-1\right)}^{K-1}} & {\left(\frac{e}{2}\right)}^{K-1+j}\frac{\left(K\right)!}{{\left(K-1\right)}^{K-1}} & \cdots & {\left(\frac{e}{2}\right)}^{K-1+j}\frac{\left(K-1+j\right)!}{{\left(K-1\right)}^{K-1}\;j^{j}} & \cdots & {\left(\frac{e}{2}\right)}^{2K-2}\frac{\left(2K-2\right)!}{{\left(K-1\right)}^{2(K-1)}} \end{array}\right]$$

*are a positive definite; matrix* Γ(α) *for an arbitrary time-scale factor* α>0*.*

The matrix $\Gamma_{1}$ is independent of the time-scale factor; only the multiplier $\frac{1}{2\alpha }$ in the last expression of (52) depends on α. Since for any symmetric non-negative definite matrix, the eigenvalues and singular values are identical, by virtue of the Rayleigh–Ritz inequalities [58] (Lemma I):

(54) $$\lambda_{min}\left(X\right)x^{T}x\leq x^{T}Xx\leq \lambda_{max}\left(X\right)x^{T}x,$$

which hold for any $x\epsilon {ℛ}^{m}$ and any symmetric matrix $X=X^{T}\epsilon {ℛ}^{m,m}$, where $\lambda_{min}\left(X\right)$ and $\lambda_{max}\left(X\right)$ are minimal and maximal eigenvalues of the matrix X, Equation (52) implies the following estimations:

$$\frac{1}{2\alpha }\sigma_{min}\left(\Gamma_{1}\right){\left\|g_{K}\right\|}_{2}^{2}\leq {\left\|H_{K}\left(\tau ,\alpha \right)\right\|}_{2}^{2}\leq \frac{1}{2\alpha }\sigma_{1}\left(\Gamma_{1}\right){\left\|g_{K}\right\|}_{2}^{2},$$

where $\sigma_{1}\left(\Gamma_{1}\right)$ and $\sigma_{min}\left(\Gamma_{1}\right)$ denote the largest and the minimal singular values of matrix $\Gamma_{1}$ (53). Thus, the next result is derived.

**Proposition** **2.** *For an arbitrary time-scale factor* α *and arbitrary vector of model parameters* $g_{K}$*, the following inequalities hold:*

(55) $$\frac{1}{\sqrt{2\alpha }}\sqrt{\sigma_{min}\left(\Gamma_{1}\right)}{\left\|g_{K}\right\|}_{2}\leq {\left\|H_{K}\left(\tau ,\alpha \right)\right\|}_{2}\leq \frac{1}{\sqrt{2\alpha }}\sqrt{\sigma_{1}\left(\Gamma_{1}\right)}{\left\|g_{K}\right\|}_{2}.$$

The square roots of the singular values $\sigma_{1}\left(\Gamma_{1}\right)$ and $\sigma_{min}\left(\Gamma_{1}\right)$ for K=5,6, …12 are summarized in Table 2. However, the lower bound of this norm is useful only for small K and small time-scale factors.

Since $\sqrt{\sigma_{1}\left(\Gamma_{1}\right)}$ grows with K, the greater the number of model summands there are, the greater the time-scaling factor should be to achieve pre-assumed multiplier $\frac{1}{\sqrt{2\alpha }}\sqrt{\sigma_{1}\left(\Gamma_{1}\right)}$ in the estimation (55). In [59], a decreasing sequence of upper bounds for the largest singular value of a non-negative definite square matrix is constructed, given by Equation (19) in [59]. This result applied to the K×K matrix $\Gamma_{1}$ means that

(56) $$\psi_{n}\left(\Gamma_{1}\right)=\frac{tr\left(\Gamma_{1}\right)}{K}+{\left[\frac{{\left(K-1\right)}^{2n-1}}{{\left(K-1\right)}^{2n-1}+1}tr\left[{\left(\Gamma_{1}-\frac{tr\left(\Gamma_{1}\right)}{K}I_{K,K}\right)}^{2n}\right]\right]}^{\frac{1}{2n}},$$

is a decreasing sequence of upper bounds for $\sigma_{1}\left(\Gamma_{1}\right)$. The right inequality in (55) can be weakened to the following:

**Proposition** **3.** *For an arbitrary time-scale factor* α *and arbitrary vector of model parameters* $g_{K}$*, the sequence of inequalities hold:*

(57) $${\left\|H_{K}\left(\tau ,\alpha \right)\right\|}_{2}\leq \frac{1}{\sqrt{2\alpha }}\sqrt{\psi_{n}\left(\Gamma_{1}\right)}{\left\|g_{K}\right\|}_{2}$$

*for* n=1,2,…*, with the coefficients* $\psi_{n}\left(\Gamma_{1}\right)$ *(56).*

In Table A2 in the Appendix B, the sequences $\sqrt{\psi_{n}\left(\Gamma_{1}\right)}$ are summarized for n=1,2,…,10 and K=4,5, …12 model summands; for comparison, in the last row, $\sqrt{\sigma_{1}\left(\Gamma_{1}\right)}$ are given. The sequence $\sqrt{\psi_{n}\left(\Gamma_{1}\right)}$ quickly decreases to $\sqrt{\sigma_{1}\left(\Gamma_{1}\right)}$; already, the sixth to eighth estimates equal $\sqrt{\sigma_{1}\left(\Gamma_{1}\right)}$. However, it should be remembered that the right inequality in (55) and (57) only give the upper bounds of the norm ${\left\|H_{K}\left(\tau ,\alpha \right)\right\|}_{2}$.

To summarize, the smoothness of the optimal solution ${\bar{g}}_{K}^{\lambda }\left(\alpha \right)$ (27) of discrete regularized problem (26) guarantees that the fluctuations in the respective spectrum of relaxation, in particular the resulting spectrum of relaxation ${\hat{H}}_{K}\left(\tau \right)$ (46), are also bounded. The time-scale factor α also affects the smoothness of the spectrum model.

#### 3.6.2. Convergence and Noise Robustness

The relaxation spectrum recovery from experimental data is an inverse ill-posed problem, in which the identification index refers to the measured relaxation modulus, but not directly to the unknown relaxation spectrum H(τ). Therefore, we cannot estimate the model error ${\left\|H\left(\tau \right)-H_{K}\left(\tau ,\alpha \right)\right\|}_{2}$ directly. As a reference point for the model $H_{K}\left(\tau ,\alpha \right)$ (6) and (27), we will consider the model of the spectrum that we would obtain for the same time-scale factor α and regularization parameter λ on the basis of ideal (undisturbed) measurements of the relaxation modulus:

(58) $${\tilde{H}}_{K}\left(\tau ,\alpha \right)=\sum_{k=0}^{K-1}g_{K}^{\lambda }\left(\alpha \right)h_{k}\left(\tau ,\alpha \right),$$

where $g_{K}^{\lambda }\left(\alpha \right)$ is the vector of the regularized solution of (26) given by (compare (27))

(59) $$g_{K}^{\lambda }\left(\alpha \right)={\left(\Phi_{N,K}^{T}\left(\alpha \right)\Phi_{N,K}\left(\alpha \right)+\lambda I_{K,K}\right)}^{-1}\Phi_{N,K}^{T}\left(\alpha \right)G_{N}$$

for noise-free measurements of relaxation modulus $G_{N}={\left[\begin{array}{ccc} G\left(t_{1}\right) & \cdots & G\left(t_{N}\right) \end{array}\right]}^{T}$; c.f., Equation (24). In Appendix A.8, the following estimations are derived:

**Proposition** **4.** *For an arbitrary time-scale factor* α *and arbitrary regularization parameter* λ*, the error between the relaxation time spectrum models* $H_{K}\left(\tau ,\alpha \right)$ *(6) and (27) and* ${\tilde{H}}_{K}\left(\tau ,\alpha \right)$ *(58) and (59) is estimated using the following inequality:*

(60) $${\left\|H_{K}\left(\tau ,\alpha \right)-{\tilde{H}}_{K}\left(\tau ,\alpha \right)\right\|}_{2}\leq \frac{1}{\sqrt{2\alpha }}\sqrt{\sigma_{1}\left(\Gamma_{1}\right)}{\left[\sum_{i=1}^{r\left(\alpha \right)}\frac{{\left[\sigma_{i}\left(\alpha \right)\right]}^{4}}{{\left\{{\left[\sigma_{i}\left(\alpha \right)\right]}^{2}+\lambda \right\}}^{4}}\right]}^{\frac{1}{4}}{\left\|z_{N}\right\|}_{2}$$

*where* $z_{N}={\left[\begin{array}{ccc} z\left(t_{1}\right) & \cdots & z\left(t_{N}\right) \end{array}\right]}^{T}$ *is the vector of measurement noises.*

According to inequality (60), the accuracy of the spectrum approximation depends both on the measurement noises and regularization parameter and on the singular values $\sigma_{1}\left(\alpha \right),\ldots ,\sigma_{r\left(\alpha \right)}\left(\alpha \right)$ of the matrix $\Phi_{N,K}\left(\alpha \right)$ (47) depending on the time-scale factor. By (60), the spectrum $H_{K}\left(\tau ,\alpha \right)$ tends to the noise-free spectrum ${\tilde{H}}_{K}\left(\tau ,\alpha \right)$ in each relaxation time τ, at which they are both continuous, linearly with respect to the norm ${\left\|z_{N}\right\|}_{2}$, as ${\left\|z_{N}\right\|}_{2}\to 0$.

### 3.7. Example

Consider a viscoelastic material of the relaxation spectrum described by the double-mode Gauss-like distribution:

(61) $$H\left(\tau \right)=\left[\beta_{1}e^{-{\left(\frac{1}{\tau }-m_{1}\right)}^{2}/q_{1}}+\beta_{2}e^{-{\left(\frac{1}{\tau }-m_{2}\right)}^{2}/q_{2}}\right]/\tau ,$$

where the parameters are as follows: $\beta_{1}=467\;\mathrm{Pa}\cdot s$, $m_{1}=0.0037{\;s}^{-1}$, $q_{1}=1.124261\times 10^{-6}{\;s}^{-2}\;$, $\beta_{2}=39\;\mathrm{Pa}\cdot s$, $m_{2}=0.045{\;s}^{-1}$ and $q_{2}=1.173\times 10^{-3}{\;s}^{-2}$. Spectra of this type are tested at the stage of developing new identification methods, for example, in [23] (Figure 2), [24] (Figures 9, 11 and 17) and [25] (Figures 2, 3, 6, 7–11 and 14), because they describe the viscoelastic properties of various materials, in particular: polymers [60] (Figures 4b and 8b), polyacrylamide gels [28] (Figure A4), glass [61] (Figure 2), wood [13] and sugar beet [18] (Figure 2). It is shown in Appendix A.9 that the corresponding ‘real’ relaxation modulus is

(62) $$G\left(t\right)=\frac{\sqrt{\pi }}{2}[\beta_{1}\sqrt{q_{1}}\;e^{\frac{1}{4}t^{2}q_{1}-m_{1}t}erfc\left(\frac{\frac{1}{2}tq_{1}-m_{1}}{\sqrt{q_{1}}}\right)+\;\beta_{2}\sqrt{q_{2}}\;e^{\frac{1}{4}t^{2}q_{2}-m_{2}t}erfc\left(\frac{\frac{1}{2}tq_{2}-m_{2}}{\sqrt{q_{2}}}\right)],$$

where the complementary error function erfc(x) is defined by [62]:

(63) $$erfc\left(x\right)=\frac{2}{\sqrt{\pi \;}\;}\int_{x}^{\infty }e^{-z^{2}}dz.$$

In the experiment, N=5000 sampling instants $t_{i}$ were generated with the constant period in the time interval T=[0,1550] seconds selected in view of the course of the modulus G(t) (62). Additive measurement noises $z\left(t_{i}\right)$ were selected independently by random choice with uniform distribution on the interval [−0.005, 0.005] Pa. The ‘real’ spectrum (61), modulus (62) and the basis functions $h_{k}\left(\tau ,\alpha \right)$, $\phi_{k}\left(t,\alpha \right)$ were simulated in Matlab R2022a using the special functions *besselk* and *erfc.* For the singular value decomposition procedure, *svd* was applied. New calculation algorithms of the modified Bessel function of the second kind are constantly being developed; recently, an algorithm for parallel calculation was proposed [63].

#### 3.7.1. Optimal Models

For K=3,4,…,12, the optimal time-scaling factors $\alpha_{opt}$ were determined via the proposed two-level identification scheme and are given in Table 3 together with the related regularization parameters $\lambda_{GCV}\left(\alpha_{opt}\right)$. Next, the vectors of optimal model parameters ${\hat{g}}_{K}={\hat{g}}_{K}^{\lambda_{GCV}}\left(\alpha_{opt}\right)$ (45) were computed and are given in Table A3 in Appendix B; the elements of these vectors are both negative and positive. The square norms ${\left\|{\hat{g}}_{K}\right\|}_{2}$ and ${\left\|H_{Kopt}\left(\tau \right)\right\|}_{2}$ are also enclosed in Table 3 as the measures of the solution smoothness. The norm ${\left\|H_{Kopt}\left(\tau \right)\right\|}_{2}$ of the optimal model $H_{Kopt}\left(\tau \right)$ (46) was determined through (52) based on ${\hat{g}}_{K}$. For the ‘real’ spectrum H(τ) (61), the norm ${\left\|H\left(\tau \right)\right\|}_{2}=19.2562\;\mathrm{Pa}$. The approximation error between H(τ) (61) and their models $H_{Kopt}\left(\tau \right)$ (46) was estimated via relative integral error ER1(K), defined by:

(64) $$ER1{\left(K\right)}^{2}=\frac{{\left\|H\left(\tau \right)-H_{Kopt}\left(\tau \right)\right\|}_{2}^{2}}{{\left\|H\left(\tau \right)\right\|}_{2}^{2}}=\frac{\int_{0}^{\infty }{\left[H\left(\tau \right)-H_{Kopt}\left(\tau \right)\right]}^{2}d\tau }{\int_{0}^{\infty }H{\left(\tau \right)}^{2}d\tau },$$

which is expressed in a percentage in the penultimate column of Table 3. For the bimodal spectrum, the values of this error of the order of 33% are not surprising in the context of the ill-conditioned inverse problem. To compare the approximations $H_{Kopt}\left(\tau \right)$ (46) obtained for successive integer K, the square index ER2(K) defined by the distance:

(65) $$ER2\left(K\right)={\left\|H_{\left(K+1\right)opt}\left(\tau \right)-H_{Kopt}\left(\tau \right)\right\|}_{2}^{2}=\int_{0}^{\infty }{\left[H_{\left(K+1\right)opt}\left(\tau \right)-H_{Kopt}\left(\tau \right)\right]}^{2}d\tau ,$$

is applied; see the last column of Table 3. The optimal indices $Q_{Nopt}\left(\alpha_{opt}\right)$ (37) are given in Table 3, too. The optimal models $H_{Kopt}\left(\tau \right)$ (46) and the ‘real’ spectrum H(τ) (61) are plotted in Figure 4 for K=3,4,…12. The analysis of the data from Table 3 and, in particular, the analysis of Figure 4b–d show that increasing the number of model components above K=8 does not significantly affect the course of the model $H_{Kopt}\left(\tau \right)$ and its accuracy. This is emphasized by the values of the ER1(K) (64) and ER2(K) (65) indices; in particular, the integral square error ER2(K) between successive $H_{Kopt}\left(\tau \right)$ and $H_{\left(K+1\right)opt}\left(\tau \right)$ spectrum approximations, which relates to the square norm ${\left\|H\left(\tau \right)\right\|}_{2}^{2}$ for K≥8 does not exceed 0.046%, and for K≥10, it is of the order of 0.009%. 

In Figure 5, the optimal models of the relaxation modulus $G_{K}\left(t,\alpha_{opt}\right)$ computed for ${\hat{g}}_{K}$ (45) according to (7) are plotted, where the measurements $\bar{G}\left(t_{i}\right)$ of the ‘real’ modulus G(t) (62) are also marked. The optimal models $G_{K}\left(t,\alpha_{opt}\right)$ have been well fitted to the experimental data, as indicated by the mean-square model errors $Q_{Nopt}\left(\alpha_{opt}\right)/N$, which for 8≤K≤12 vary in the range from $4.889\cdot 10^{-9}\;{\mathrm{Pa}}^{2}$ to $5.0482\cdot 10^{-9}\;{\mathrm{Pa}}^{2}$. Thus, models $G_{K}\left(t,\alpha_{opt}\right)$ for different K practically coincide with the measurement points and with each other; see Figure 5.

#### 3.7.2. Optimal and Sub-Optimal Time-Scale Factors

The identification index $Q_{Nopt}\left(\alpha \right)$ (35) minimized in the upper-level task (37) as a function of time-scale factor α is plotted in Figure 6a–d for K=6,8,10,12. The optimal parameters $\alpha_{opt}$ are marked. The analysis of these plots suggests that there is such a neighborhood of $\alpha_{opt}$, namely a closed interval $\left[\alpha_{min},\alpha_{max}\right]$, that $\alpha_{min}\leq \alpha_{opt}\leq \alpha_{max}$ and for each $\alpha \in \left[\alpha_{min},\alpha_{max}\right]$, the identification index $Q_{Nopt}\left(\alpha \right)$ differs from the minimal $Q_{Nopt}\left(\alpha_{opt}\right)$ not more than $\varepsilon_{\alpha }\cdot Q_{Nopt}\left(\alpha_{opt}\right)$, i.e.,

(66) $$Q_{Nopt}\left(\alpha \right)-Q_{Nopt}\left(\alpha_{opt}\right)\leq \varepsilon_{\alpha }\cdot Q_{Nopt}\left(\alpha_{opt}\right),$$

where $\varepsilon_{\alpha }$ is a small positive number. Inequality (66) means the deterioration of the model error is not greater than $\varepsilon_{\alpha }$ percent of the optimal $Q_{Nopt}\left(\alpha_{opt}\right).$ Any parameter α from the interval $\left[\alpha_{min},\alpha_{max}\right]$ is a suboptimal time-scale factor. In Figure 6, $\varepsilon_{\alpha }=0.1$, which means a 10% level of sub-optimality, is assumed, and the values of $\alpha_{min}$ and $\alpha_{max}$ are marked on the small subfigures. They are also given in Table 3. With the increase in the number of model components, the optimal parameter $\alpha_{opt}$ increases and the range of sub-optimal time-scale factors shifts. For an exemplary number of model components, K=9, the optimal model $H_{Kopt}\left(\tau \right)$ (46) and models $H_{K}\left(\tau ,\alpha \right)$ (6), optimal in the sense of lower-level task (28) for suboptimal parameters $\alpha_{min}$ and $\alpha_{max}$, are plotted in Figure 7a; the ‘real’ spectrum H(τ) (61) is presented, too. The corresponding optimal regularization parameters are as follows: $\lambda_{GCV}\left(\alpha_{min}\right)=0.0167$ and $\lambda_{GCV}\left(\alpha_{max}\right)=0.0094$. For respective vectors of optimal model parameters, we have ${\left\|{\hat{g}}_{K}^{\lambda_{GCV}}\left(\alpha_{min}\right)\right\|}_{2}=2.5114\;\mathrm{Pa}$ and ${\left\|{\hat{g}}_{K}^{\lambda_{GCV}}\left(\alpha_{max}\right)\right\|}_{2}=2.5119\;\mathrm{Pa}$. The norms of the relaxation spectra are ${\left\|H_{K}\left(\tau ,\alpha_{min}\right)\right\|}_{2}=17.4472\;\mathrm{Pa}$ and ${\left\|H_{K}\left(\tau ,\alpha_{max}\right)\right\|}_{2}=20.2069\;\mathrm{Pa}$. In Figure 7b, the ‘real’ modulus G(t) (62) and the models: $G_{K}\left(t,\alpha_{opt}\right)$ (7) and (45) and $G_{K}\left(t,\alpha_{min}\right)$, $G_{K}\left(t,\alpha_{max}\right)$ computed for ${\hat{g}}_{K}^{\lambda_{GCV}}\left(\alpha_{min}\right)$, ${\hat{g}}_{K}^{\lambda_{GCV}}\left(\alpha_{max}\right)$ according to (7) are plotted. However, the relaxation moduli are almost identical (Figure 7b), the spectrum models differ (Figure 7a), which emphasizes the importance of the time-scale factor optimal selection. Too strong smoothing of the relaxation spectrum models, with a simultaneous very good fit to the experimental data of the relaxation modulus models, indicates the need to modify the quality index in the regularized task (26). For example, the square term ${\|g_{K}\|}_{2}^{2}$ in the second component of the objective function can be replaced by the quadratic form $g_{K}^{T}Wg_{K}$, and a positive definite weight matrix W or regularized weighted least-squares approach [41] can be applied. This will be the subject of further work.

## 4. Conclusions

In this paper, a new robust hierarchical algorithm for the identification of the relaxation time spectrum, which combines the technique of an expansion of a function into a series of basis functions with the least-squares regularized identification and the optimal choice of the time-scale factor, has been derived. The task of determining the best ‘regularized’ model was solved at the lower level, while the optimal time-scale factor was selected on the upper level of the identification scheme. The continuous spectrum of relaxation times was approximated by finite series of power–exponential basis functions, while the components of the relaxation modulus model were proven to be described by the product of power of time and the modified Bessel function of the second kind. In the present paper, the problem of the optimal choice of the time-scale factor to ensure the best fit of the model to experimental data has been formulated and solved for the first time in the context of the relaxation spectrum identification.

The necessary optimality conditions both for the optimal regularized least-squares identification task and the problem of the optimal selection of the time-scale factor were derived in the form of nonlinear algebraic equations. The main properties of the basis functions of the relaxation spectrum and modulus models, their positive definiteness, convenient upper bounds, monotonicity, asymptotic properties and wide range of applicability for different time-scale factors indicated the possibility of using the proposed model and identification algorithm to determine the spectrum of a wide class of viscoelastic materials.

The overly strong smoothing of the relaxation spectrum models in the example, with a very good fit to the experimental data of the relaxation modulus models, indicates the need to modify the quality index in the lower level identification task. An introduction of a weight matrix in the second component of the objective function or a direct application of regularized weighted least-squares should be investigated. Another solution is the selection of such a spectrum model which guarantees the assumed level of smoothing and optimal adjustment to the relaxation modulus measurement data. These approaches will be the subject of further work.

The presented scheme of the relaxation spectrum identification can be easily modified for retardation spectrum recovery from creep compliance measurements obtained in the standard creep test.

## Figures and Tables

**Table 1:** Ranges of the applicability of the model for various time-scale parameters for K=5 and K=12.

Time-Scale Factor *α* [s]	K=5	K=5	K=12	K=12
	Range ^1^ of Relaxation Times *τ_app_*(*α*) [s]	Range ^1^ of Times *t_app_*(*α*) [s]	Range ^1^ of Relaxation Times *τ_app_*(*α*) [s]	Range ^1^ of Times *t_app_*(*α*) [s]
0.0001	144,305.22	282,360.6	255,824.35	662,077.1
0.001	14,430.52	28,236.06	25,582.435	66,207.71
0.01	1443.05	2823.61	2558.243	6620.77
0.1	144.305	282.36	255.824	662.08
1	14.43	28.236	25.5824	66.208
10	1.4431	2.824	2.5582	6.621
100	0.1443	0.282	0.2558	0.662

^1^ The upper bounds $t_{app}$ (20) and $\tau_{app}\left(\alpha \right)$ (21) of the applicability intervals $\left[0,t_{app}\left(\alpha \right)\right]$ and $\left[0,\tau_{app}\left(\alpha \right)\right]$ are given.

**Table 2:** The square roots of the largest $\sigma_{1}\left(\Gamma_{1}\right)$ and minimal $\sigma_{min}\left(\Gamma_{1}\right)$ singular values of the matrix $\Gamma_{1}$ (53) for K=4,5, …12 model summands.

*K*	4	5	6	7	8	9	10	11	12
$\sqrt{\sigma_{1}\left(\Gamma_{1}\right)}$	3.666396	4.325185	4.927061	5.485792	6.010247	6.506534	6.979079	7.431223	7.865567
$\sqrt{\sigma_{min}\left(\Gamma_{1}\right)}$	0.206481	0.087523	0.034642	0.013243	0.004953	0.001824	6.6358 × 10^−4^	2.3925 × 10^−4^	8.5615 × 10^−5^

**Table 3:** The parameters of the optimal models in the example: optimal time-scale factors $\alpha_{opt}$, upper $\alpha_{min}$ and lower $\alpha_{max}$ bounds of the interval $\left[\alpha_{min},\alpha_{max}\right]$ of suboptimal time-scale factors defined by inequality (66) for $\varepsilon_{\alpha }=0.1$, regularization parameters $\lambda_{GCV}\left(\alpha_{opt}\right)$, optimal identification indices $Q_{Nopt}\left(\alpha_{opt}\right)$ defined in (37), square norms: ${\left\|{\hat{g}}_{K}\right\|}_{2}$ of the vector of optimal model parameter ${\hat{g}}_{K}$ (45) and ${\left\|H_{Kopt}\left(\tau \right)\right\|}_{2}$ of the optimal relaxation spectrum model $H_{Kopt}\left(\tau \right)$ (46), relaxation spectrum approximation error ER1(K) defined in (64) and the distance between successive approximations $H_{Kopt}\left(\tau \right)$ measured by ER2(K) (65).

K	$\alpha_{opt}$ $\left[s^{-1}\right]$	$\alpha_{min}$ $\left[s^{-1}\right]$	$\alpha_{max}$ $\left[s^{-1}\right]$	$\lambda_{GCV}\left(\alpha_{opt}\right)$ [−]	$Q_{Nopt}\left(\alpha_{opt}\right)$ $\left[{\mathrm{Pa}}^{2}\right]$	${\left\|{\hat{g}}_{K}\right\|}_{2}$ [Pa]	${\left\|H_{Kopt}\left(\tau \right)\right\|}_{2}$ [Pa]	ER1(K) [%]	ER2(K) $\left[{\mathrm{Pa}}^{2}\cdot s\right]$
3	0.00520	0.00205	0.0080	0.0445	8.63505 × 10^−4^	0.7055	13.0248	90.459	12.876
4	0.01675	0.0164	0.01745	0.0063	3.43945 × 10^−5^	7.3485	16.1607	39.160	3.1049
5	0.02025	0.0192	0.0213	0.0071	2.71552 × 10^−5^	4.6724	17.1465	35.638	2.4592
6	0.02375	0.0220	0.0255	0.0078	2.48511 × 10^−5^	3.6493	18.0443	33.986	0.7563
7	0.02655	0.0234	0.0290	0.0083	2.48256 × 10^−5^	2.8846	18.5088	33.364	0.1432
8	0.02865	0.0234	0.0318	0.0089	2.51617 × 10^−5^	2.3555	18.5679	32.824	0.1692
9	0.03005	0.0241	0.03425	0.0099	2.52412 × 10^−5^	2.0639	18.3756	32.701	0.0111
10	0.03215	0.0255	0.0367	0.0109	2.51143 × 10^−5^	1.9058	18.4224	32.631	0.0336
11	0.03390	0.0276	0.03915	0.0122	2.48521 × 10^−5^	1.8020	18.3498	32.879	0.0327
12	0.03670	0.02935	0.04195	0.0127	2.44452 × 10^−5^	1.7198	18.4432	32.919	0.0328

## Data Availability

Not applicable.

## Appendix A

### Appendix A.1. Proof of Theorem 1

To prove the theorem, we first derive the following integral formula:

(A1) $$ℐ=\int_{0}^{\infty }\tau^{v-1}e^{-\alpha \tau }e^{-t/\tau }d\tau =2{\left(\sqrt{\frac{t}{\alpha }}\right)}^{v}K_{v}\left(2\sqrt{\alpha t}\right),$$

where v∈ℛ.

By applying in the integral of the left-hand side of (A1) the substitution $\sqrt{\alpha }\;\tau =\sqrt{t}e^{y}$ we have:

$$ℐ=\int_{-\infty }^{\infty }{\left(\sqrt{\frac{t}{\alpha }}\right)}^{v}e^{y\left(v-1\right)}e^{-\sqrt{\alpha t}\;e^{y}}e^{-\sqrt{\alpha t}\;e^{-y}}e^{y}dy,$$

which can be expressed as

(A2) $$ℐ={\left(\sqrt{\frac{t}{\alpha }}\right)}^{v}\int_{-\infty }^{\infty }e^{vy}e^{-2\sqrt{\alpha t}\;cosh\left(y\right)}dy.$$

Since, bearing in mind that cosh(y) is an even function, the integral in (A2) is rewritten as

$$ℐ={\left(\sqrt{\frac{t}{\alpha }}\right)}^{v}\left[\int_{0}^{\infty }e^{-vy\;}e^{-2\sqrt{\alpha t}\;cosh\left(y\right)}dy+\int_{0}^{\infty }e^{vy\;}e^{-2\sqrt{\alpha t}\;cosh\left(y\right)}dy\right],$$

or equivalently

$$ℐ=2{\left(\sqrt{\frac{t}{\alpha }}\right)}^{v}\int_{0}^{\infty }e^{-2\sqrt{\alpha t}\;cosh\left(y\right)}cosh\left(vy\right)dy,$$

whence, by the following integral representation formula for the Bessel functions [45,46] (Equation (5) on p. 181 in [46]):

$$K_{v}\left(x\right)=\int_{0}^{\infty }e^{-x\;cosh\left(y\right)}cosh\left(vy\right)dy,$$

where x>0 and v∈ℛ, Formula (A1) directly follows.

Now, we prove Formula (9). Since, by (3) and (8) the basis functions

$$\phi_{k}\left(t,\alpha \right)={\left(\frac{\alpha }{k}\right)}^{k}e^{k}\int_{0}^{\infty }\tau^{k-1}e^{-\alpha \tau }e^{-t/\tau }d\tau$$

from (A1), we have

$$\phi_{k}\left(t,\alpha \right)=2{\left(\frac{\alpha }{k}\right)}^{k}e^{k}{\left(\sqrt{\frac{t}{\alpha }}\right)}^{k}K_{k}\left(2\sqrt{\alpha t}\right),\;$$

whence, Formula (9) follows. □

### Appendix A.2. Algebraic Matrix Properties

In this appendix, a new differential matrix property, used to obtain the optimality conditions and to simplify numerical computations, is derived. First, for convenience, some matrix identities and inequalities known in the literature are also provided.

#### Appendix A.2.1. Matrix Identities and Inequalities

The following identities hold for arbitrary matrices A, B**,** C and D **[57]** (Equations (6.4.2)), (2.1.1) and (2.1.4), in order:

(A3) tr[AB]=tr[BA], 

(A4) $${\left(AB\right)}^{-1}=B^{-1}A^{-1},\;$$

(A5) $${\left(BC+A\right)}^{-1}=A^{-1}-A^{-1}B{\left(I+CA^{-1}B\right)}^{-1}CA^{-1},\;$$

provided that respective matrices are invertible.

The following differential properties hold for arbitrary differentiable matrix functions A(x) and B(x) [57] (Equations (P2.1.2a) and (P2.1.2b), respectively):

(A6) $$\frac{\partial }{\partial x}\left[A\left(x\right)B\left(x\right)\right]=\frac{\partial A\left(x\right)}{\partial x}B\left(x\right)+A\left(x\right)\frac{\partial B\left(x\right)}{\partial x},\;$$

(A7) $$\frac{\partial }{\partial x}\left[A{\left(x\right)}^{-1}\right]=-A{\left(x\right)}^{-1}\frac{\partial A\left(x\right)}{\partial x}A{\left(x\right)}^{-1},\;$$

assuming that matrix A(x) is invertible.

#### Appendix A.2.2. New Matrix Property

Let us now prove the following result.

**Proposition** **A1.** *Let* A(x) *be a symmetric positive definite* m×m *matrix function differentiable with respect to the real argument* x*. Then*

(A8) $$\frac{\partial tr\left[A{\left(x\right)}^{-1}\right]}{\partial x}=-tr\left[A{\left(x\right)}^{-1}\frac{\partial A\left(x\right)}{\partial x}A{\left(x\right)}^{-1}\right].$$

**Proof of Proposition** **A1.** Since the trace of the matrix is a linear function, bearing in mind definition of the matrix derivative with respect to the scalar argument x [57] (Section 2.1.8), we have

$$\frac{\partial tr\left[A{\left(x\right)}^{-1}\right]}{\partial x}=tr\left[\frac{\partial }{\partial x}\left[A{\left(x\right)}^{-1}\right]\right],$$

which, in view of (A7), directly yields (A8); the proposition is proved. □

### Appendix A.3. Proof of Theorem 2

First, we express Ξ(λ,α) (30) in a more useful equivalent form. By the matrix identity (A5) for λ>0, we have

$$\lambda {\left(\Phi_{N,K}\left(\alpha \right)\Phi_{N,K}^{T}\left(\alpha \right)+\lambda I_{N,N}\right)}^{-1}=I_{N,N}-\Phi_{N,K}\left(\alpha \right)(\lambda I_{K,K}+\;{\Phi_{N,K}^{T}\left(\alpha \right)\Phi_{N,K}\left(\alpha \right))}^{-1}\Phi_{N,K}^{T}\left(\alpha \right),$$

whereas, by (30) and bearing in mind the notation (32), we obtain

(A9) $$\Xi \left(\lambda ,\alpha \right)=\lambda {\left(\Psi \left(\alpha \right)+\lambda I_{N,N}\right)}^{-1}.$$

Since, by (30), the GCV functional $V_{GCV}\left(\lambda ,\alpha \right)$ (29) is expressed as

$$V_{GCV}\left(\lambda ,\alpha \right)=\frac{{\bar{G}}_{N}^{T}\Xi \left(\lambda ,\alpha \right)\Xi \left(\lambda ,\alpha \right){\bar{G}}_{N}}{tr{\left[\Xi \left(\lambda ,\alpha \right)\right]}^{2}},$$

its derivative with respect to λ is given by

(A10) $$\frac{\partial V_{GCV}\left(\lambda ,\alpha \right)}{\partial \lambda }=\frac{\frac{\partial }{\partial \lambda }\left[{\bar{G}}_{N}^{T}\Xi \left(\lambda ,\alpha \right)\Xi \left(\lambda ,\alpha \right){\bar{G}}_{N}\right]tr\left[\Xi \left(\lambda ,\alpha \right)\right]-2{\bar{G}}_{N}^{T}\Xi \left(\lambda ,\alpha \right)\Xi \left(\lambda ,\alpha \right){\bar{G}}_{N}\frac{\partial tr\left[\Xi \left(\lambda ,\alpha \right)\right]}{\partial \lambda }}{tr{\left[\Xi \left(\lambda ,\alpha \right)\right]}^{3}}.$$

Now, we determine the partial derivatives which appear in the nominator of the right-hand side of (A10). By Formula (A6), we have

(A11) $$\frac{\partial }{\partial \lambda }\left[{\bar{G}}_{N}^{T}\Xi \left(\lambda ,\alpha \right)\Xi \left(\lambda ,\alpha \right){\bar{G}}_{N}\right]=2{\bar{G}}_{N}^{T}\Xi \left(\lambda ,\alpha \right)\frac{\partial \Xi \left(\lambda ,\alpha \right)}{\partial \lambda }{\bar{G}}_{N}.$$

Equation (A9) yields

(A12) $$\frac{\partial \Xi \left(\lambda ,\alpha \right)}{\partial \lambda }={\left(\Psi \left(\alpha \right)+\lambda I_{N,N}\right)}^{-1}+\lambda \frac{\partial }{\partial \lambda }\left[{\left(\Psi \left(\alpha \right)+\lambda I_{N,N}\right)}^{-1}\right],$$

where the derivative of the inverse matrix can be found due to property (A7), which, when applied to (A12), results in

$$\frac{\partial \Xi \left(\lambda ,\alpha \right)}{\partial \lambda }={\left(\Psi \left(\alpha \right)+\lambda I_{N,N}\right)}^{-1}-\lambda {\left(\Psi \left(\alpha \right)+\lambda I_{N,N}\right)}^{-1}{\left(\Psi \left(\alpha \right)+\lambda I_{N,N}\right)}^{-1},$$

whereas, after algebraic manipulations, we obtain

(A13) $$\frac{\partial \Xi \left(\lambda ,\alpha \right)}{\partial \lambda }={\left(\Psi \left(\alpha \right)+\lambda I_{N,N}\right)}^{-2}\Psi \left(\alpha \right).$$

Combining (A11), (A13) and (A9) yields

(A14) $$\frac{\partial }{\partial \lambda }\left[{\bar{G}}_{N}^{T}\Xi \left(\lambda ,\alpha \right)\Xi \left(\lambda ,\alpha \right){\bar{G}}_{N}\right]=2\lambda {\bar{G}}_{N}^{T}{\left(\Psi \left(\alpha \right)+\lambda I_{N,N}\right)}^{-3}\Psi \left(\alpha \right){\bar{G}}_{N}.$$

To find $\frac{\partial tr\left[\Xi \left(\lambda ,\alpha \right)\right]}{\partial \lambda }$, expression (A9) is used, which implies

(A15) $$tr\left[\Xi \left(\lambda ,\alpha \right)\right]=\lambda \;tr\left[{\left(\Psi \left(\alpha \right)+\lambda I_{N,N}\right)}^{-1}\right],$$

whence

(A16) $$\frac{\partial tr\left[\Xi \left(\lambda ,\alpha \right)\right]}{\partial \lambda }=tr\left[{\left(\Psi \left(\alpha \right)+\lambda I_{N,N}\right)}^{-1}\right]+\lambda \;\frac{\partial tr\left[{\left(\Psi \left(\alpha \right)+\lambda I_{N,N}\right)}^{-1}\right]}{\partial \lambda }.$$

By Proposition A1 and Equation (A8), we have

$$\frac{\partial tr\left[{\left(\Psi \left(\alpha \right)+\lambda I_{N,N}\right)}^{-1}\right]}{\partial \lambda }=-tr\left[{\left(\Psi \left(\alpha \right)+\lambda I_{N,N}\right)}^{-2}\right];$$

therefore, derivative $\frac{\partial tr\left[\Xi \left(\lambda ,\alpha \right)\right]}{\partial \lambda }$ (A16) takes the form

(A17) $$\frac{\partial tr\left[\Xi \left(\lambda ,\alpha \right)\right]}{\partial \lambda }=tr\left[{\left(\Psi \left(\alpha \right)+\lambda I_{N,N}\right)}^{-1}\right]-\lambda \;tr\left[{\left(\Psi \left(\alpha \right)+\lambda I_{N,N}\right)}^{-2}\right].$$

Now, we can derive the necessary optimality condition. Since, in view of (A9), matrix Ξ(λ,α) is positive definite for any λ>0, derivative $\frac{\partial V_{GCV}\left(\lambda ,\alpha \right)}{\partial \lambda }$ given by the fraction from the right-hand side of (A10) is equal to zero, if and only if its nominator is equal zero to, i.e.,

(A18) $$\frac{\partial }{\partial \lambda }\left[{\bar{G}}_{N}^{T}\Xi \left(\lambda ,\alpha \right)\Xi \left(\lambda ,\alpha \right){\bar{G}}_{N}\right]tr\left[\Xi \left(\lambda ,\alpha \right)\right]=2{\bar{G}}_{N}^{T}\Xi \left(\lambda ,\alpha \right)\Xi \left(\lambda ,\alpha \right){\bar{G}}_{N}\frac{\partial tr\left[\Xi \left(\lambda ,\alpha \right)\right]}{\partial \lambda }.$$

By (A18), (A14), (A9), and (A17), we have

$$2\lambda^{2}{\bar{G}}_{N}^{T}{\left(\Psi \left(\alpha \right)+\lambda I_{N,N}\right)}^{-3}\Psi \left(\alpha \right){\bar{G}}_{N}tr\left[{\left(\Psi \left(\alpha \right)+\lambda I_{N,N}\right)}^{-1}\right]=\;2\lambda^{2}{\bar{G}}_{N}^{T}{\left(\Psi \left(\alpha \right)+\lambda I_{N,N}\right)}^{-2}{\bar{G}}_{N}\left\{tr\left[{\left(\Psi \left(\alpha \right)+\lambda I_{N,N}\right)}^{-1}\right]-\lambda \;tr\left[{\left(\Psi \left(\alpha \right)+\lambda I_{N,N}\right)}^{-2}\right]\right\},$$

which is equivalent to

(A19) $${\bar{G}}_{N}^{T}{\left(\Psi \left(\alpha \right)+\lambda I_{N,N}\right)}^{-3}\Psi \left(\alpha \right){\bar{G}}_{N}tr\left[{\left(\Psi \left(\alpha \right)+\lambda I_{N,N}\right)}^{-1}\right]={\bar{G}}_{N}^{T}(\Psi \left(\alpha \right)+\;{\lambda I_{N,N})}^{-2}{\bar{G}}_{N}\left\{tr\left[{\left(\Psi \left(\alpha \right)+\lambda I_{N,N}\right)}^{-1}\right]-\lambda \;tr\left[{\left(\Psi \left(\alpha \right)+\lambda I_{N,N}\right)}^{-2}\right]\right\},$$

whence, by standard algebraic manipulations, Equation (31) follows directly. The theorem is proved. □

### Appendix A.4. Derivation of the Formula (33)

To derive the Formula (33) describing the derivative $\frac{\partial V_{GCV}\left(\lambda ,\alpha \right)}{\partial \lambda }$ directly as a function of the matrices Ψ(α) and ${\bar{G}}_{N}$, let us consider the nominator of the right-hand side of (A10), i.e., the function

$$M\left(\lambda ,\alpha \right)=\frac{\partial }{\partial \lambda }\left[{\bar{G}}_{N}^{T}\Xi \left(\lambda ,\alpha \right)\Xi \left(\lambda ,\alpha \right){\bar{G}}_{N}\right]tr\left[\Xi \left(\lambda ,\alpha \right)\right]-\;2{\bar{G}}_{N}^{T}\Xi \left(\lambda ,\alpha \right)\Xi \left(\lambda ,\alpha \right){\bar{G}}_{N}\frac{\partial tr\left[\Xi \left(\lambda ,\alpha \right)\right]}{\partial \lambda },$$

which, by (A14), (A15), (A9) and (A17), is expressed as

$$M\left(\lambda ,\alpha \right)=2\lambda^{2}{\bar{G}}_{N}^{T}{\left(\Psi \left(\alpha \right)+\lambda I_{N,N}\right)}^{-3}\Psi \left(\alpha \right){\bar{G}}_{N}\;tr\left[{\left(\Psi \left(\alpha \right)+\lambda I_{N,N}\right)}^{-1}\right]-\;2\lambda^{2}{\bar{G}}_{N}^{T}{\left(\Psi \left(\alpha \right)+\lambda I_{N,N}\right)}^{-2}{\bar{G}}_{N}\left\{tr\left[{\left(\Psi \left(\alpha \right)+\lambda I_{N,N}\right)}^{-1}\right]-\lambda \;tr\left[{\left(\Psi \left(\alpha \right)+\lambda I_{N,N}\right)}^{-2}\right]\right\}.$$

Through tedious algebraic manipulations, the above expression is rewritten as

(A20) $$M\left(\lambda ,\alpha \right)=-2\lambda^{3}{\bar{G}}_{N}^{T}{\left(\Psi \left(\alpha \right)+\lambda I_{N,N}\right)}^{-3}\;{\bar{G}}_{N}tr\left[{\left(\Psi \left(\alpha \right)+\lambda I_{N,N}\right)}^{-1}\right]+\;2\lambda^{3}{\bar{G}}_{N}^{T}{\left(\Psi \left(\alpha \right)+\lambda I_{N,N}\right)}^{-2}{\bar{G}}_{N}tr\left[{\left(\Psi \left(\alpha \right)+\lambda I_{N,N}\right)}^{-2}\right].$$

Now, (A10) combined with (A20) and (A15) yields Formula (33).

### Appendix A.5. Proof of Theorem 3

We first prove Equation (39) for k=0; next, Formula (38) is derived.

Based on (10), property (19) and bearing in mind that $K_{1}\left(x\right)=K_{-1}\left(x\right)$ [54], we have

(A21) $$\frac{d}{d\alpha }\left[\phi_{0}\left(t,\alpha \right)\right]=-\frac{\sqrt{t}}{\sqrt{\alpha }}\left[K_{-1}\left(2\sqrt{\alpha t}\right)+K_{1}\left(2\sqrt{\alpha t}\right)\right]=-2\frac{\sqrt{t}}{\sqrt{\alpha }}K_{1}\left(2\sqrt{\alpha t}\right)\;,$$

whence, by (9) and the last equation in (A21), we immediately obtain Equation (39).

Since, for an arbitrary k≥1, the basis function $\phi_{k}\left(t,\alpha \right)$ (9) is expressed as

$$\phi_{k}\left(t,\alpha \right)=2{\left(\frac{e}{2k}\right)}^{k}{\left(2\sqrt{\alpha t}\right)}^{k}K_{k}\left(2\sqrt{\alpha t}\right)\;,$$

by Formula (18), we immediately have

$$\frac{d}{d\alpha }\left[\phi_{k}\left(t,\alpha \right)\right]=-2\frac{\sqrt{t}}{\sqrt{\alpha }}e^{k}{\left(\frac{1}{2k}\right)}^{k}{\left(2\sqrt{\alpha t}\right)}^{k}K_{k-1}\left(2\sqrt{\alpha t}\right),$$

which is rewritten as

$$\frac{d}{d\alpha }\left[\phi_{k}\left(t,\alpha \right)\right]=-2e\left(\frac{t}{k}\right){\left(\frac{k-1}{k}\right)}^{k-1}e^{k-1}{\left(\frac{\sqrt{\alpha t}}{k-1}\right)}^{k-1}K_{k-1}\left(2\sqrt{\alpha t}\right),$$

whence, in view of (9), Formula (38) follows directly, which completes the proof. □

### Appendix A.6. Proof of Theorem 4

Let us find the derivative of the function $Q_{Nopt}\left(\alpha \right)$. Based on (36) and property (A6), and bearing in mind definition (42), we have

$$\frac{dQ_{Nopt}\left(\alpha \right)}{d\alpha }=2\lambda_{GCV}\left(\alpha \right)\frac{d\lambda_{GCV}\left(\alpha \right)}{d\alpha }{\bar{G}}_{N}^{T}\Upsilon {\left(\alpha \right)}^{-2}{\bar{G}}_{N}+\;2\lambda_{GCV}^{2}\left(\alpha \right){\bar{G}}_{N}^{T}\left[\frac{d}{d\alpha }\Upsilon {\left(\alpha \right)}^{-1}\right]\Upsilon {\left(\alpha \right)}^{-1}{\bar{G}}_{N},$$

whereas by property (A7), the next formula follows

(A22) $$\frac{dQ_{Nopt}\left(\alpha \right)}{d\alpha }=2\left[\lambda_{GCV}\left(\alpha \right)\frac{d\lambda_{GCV}\left(\alpha \right)}{d\alpha }{\bar{G}}_{N}^{T}-\lambda_{GCV}^{2}\left(\alpha \right){\bar{G}}_{N}^{T}\Upsilon {\left(\alpha \right)}^{-1}\left[\frac{d}{d\alpha }\Upsilon \left(\alpha \right)\right]\right]\Upsilon {\left(\alpha \right)}^{-2},$$

where, by (42):

(A23) $$\frac{d}{d\alpha }\Upsilon \left(\alpha \right)=\frac{d}{d\alpha }\Psi \left(\alpha \right)+\frac{d\lambda_{GCV}\left(\alpha \right)}{d\alpha }I_{N,N}=\Omega_{N,K}\left(\alpha \right)+\frac{d\lambda_{GCV}\left(\alpha \right)}{d\alpha }I_{N,N}.$$

Substituting (A23) into (A22), we obtain

(A24) $$\frac{dQ_{Nopt}\left(\alpha \right)}{d\alpha }=2\lambda_{GCV}\left(\alpha \right)\frac{d\lambda_{GCV}\left(\alpha \right)}{d\alpha }{\bar{G}}_{N}^{T}\Upsilon {\left(\alpha \right)}^{-2}{\bar{G}}_{N}-\;2\lambda_{GCV}^{2}\left(\alpha \right)\frac{d\lambda_{GCV}\left(\alpha \right)}{d\alpha }{\bar{G}}_{N}^{T}\Upsilon {\left(\alpha \right)}^{-3}{\bar{G}}_{N}-2\lambda_{GCV}^{2}\left(\alpha \right){\bar{G}}_{N}^{T}\Upsilon {\left(\alpha \right)}^{-1}\Omega_{N,K}\left(\alpha \right)\Upsilon {\left(\alpha \right)}^{-2}{\bar{G}}_{N}.$$

Whence, if the parameter $\alpha_{opt}$ solves the minimization task (37), then $\lambda_{GCV}\left(\alpha_{opt}\right)$ is a solution of the algebraic Equation (40), resulting directly from equating the derivative $\frac{dQ_{Nopt}\left(\alpha \right)}{d\alpha }$ (A24) to zero. The inspection of (40) shows that two derivatives, $\Omega_{N,K}\left(\alpha \right)=\frac{d}{d\alpha }\Psi \left(\alpha \right)$ introduced in (A23) and $\frac{d\lambda_{GCV}\left(\alpha \right)}{d\alpha }$, are necessary to define (40) well.

By virtue of (32) and (A6), we obtain

(A25) $$\frac{d}{d\alpha }\Psi \left(\alpha \right)=\frac{d}{d\alpha }\Phi_{N,K}\left(\alpha \right)\Phi_{N,K}^{T}\left(\alpha \right)+\Phi_{N,K}\left(\alpha \right)\frac{d}{d\alpha }\Phi_{N,K}^{T}\left(\alpha \right).$$

Derivative $\frac{d}{d\alpha }\Phi_{N,K}\left(\alpha \right)$ of the matrix $\Phi_{N,K}\left(\alpha \right)$ follows from Theorem 3 and the structure of $\Phi_{N,K}\left(\alpha \right)$ (24). By (24), (38) and (39), bearing in mind that $0^{0}=1$, matrix $\Theta_{N,K}\left(\alpha \right)=\frac{d}{d\alpha }\Phi_{N,K}\left(\alpha \right)$ is given by (44). Thus, $\frac{d}{d\alpha }\Psi \left(\alpha \right)=\Omega_{N,K}\left(\alpha \right)$ (A25) is expressed by (43).

The regularization parameter $\lambda_{GCV}\left(\alpha \right)$, which is defined by the minimization task (28), satisfies the necessary condition (31) from Theorem 2; thus, to evaluate derivative $\frac{d\lambda_{GCV}\left(\alpha \right)}{d\alpha }$, Equation (31) is used. By (31), bearing in mind the notation (42), we have

$${\bar{G}}_{N}^{T}\Upsilon {\left(\alpha \right)}^{-3}{\bar{G}}_{N}tr\left[\Upsilon {\left(\alpha \right)}^{-1}\right]={\bar{G}}_{N}^{T}\Upsilon {\left(\alpha \right)}^{-2}{\bar{G}}_{N}\;tr\left[\Upsilon {\left(\alpha \right)}^{-2}\right].$$

Differentiating both sides of the above equation with respect to α, and bearing in mind the properties (A7) and (A8), we obtain

(A26) $${\bar{G}}_{N}^{T}\Upsilon {\left(\alpha \right)}^{-3}\left[\frac{d}{d\alpha }\Upsilon {\left(\alpha \right)}^{3}\right]\Upsilon {\left(\alpha \right)}^{-3}{\bar{G}}_{N}tr\left[\Upsilon {\left(\alpha \right)}^{-1}\right]+{\bar{G}}_{N}^{T}\Upsilon {\left(\alpha \right)}^{-3}{\bar{G}}_{N}tr\left[\Upsilon {\left(\alpha \right)}^{-1}\frac{d}{d\alpha }\Upsilon \left(\alpha \right)\Upsilon {\left(\alpha \right)}^{-1}\right]=\;{\bar{G}}_{N}^{T}\Upsilon {\left(\alpha \right)}^{-2}\left[\frac{d}{d\alpha }\Upsilon {\left(\alpha \right)}^{2}\right]\Upsilon {\left(\alpha \right)}^{-2}{\bar{G}}_{N}\;tr\left[\Upsilon {\left(\alpha \right)}^{-2}\right]+{\bar{G}}_{N}^{T}\Upsilon {\left(\alpha \right)}^{-2}{\bar{G}}_{N}\;tr\left[\Upsilon {\left(\alpha \right)}^{-2}\frac{d}{d\alpha }\Upsilon {\left(\alpha \right)}^{2}\Upsilon {\left(\alpha \right)}^{-2}\right],$$

where derivative $\frac{d}{d\alpha }\Upsilon \left(\alpha \right)$ is given by (A23). By (42) and property (A6), we have

(A27) $$\frac{d}{d\alpha }\Upsilon {\left(\alpha \right)}^{2}=\left[\frac{d}{d\alpha }\Upsilon \left(\alpha \right)\right]\Upsilon \left(\alpha \right)+\Upsilon \left(\alpha \right)\frac{d}{d\alpha }\Upsilon \left(\alpha \right),$$

and

$$\frac{d}{d\alpha }\Upsilon {\left(\alpha \right)}^{3}=\left[\frac{d}{d\alpha }\Upsilon \left(\alpha \right)\right]\Upsilon {\left(\alpha \right)}^{2}+\Upsilon \left(\alpha \right)\frac{d}{d\alpha }\Upsilon {\left(\alpha \right)}^{2},$$

whence

(A28) $$\frac{d}{d\alpha }\Upsilon {\left(\alpha \right)}^{3}=\left[\frac{d}{d\alpha }\Upsilon \left(\alpha \right)\right]\Upsilon {\left(\alpha \right)}^{2}+\Upsilon \left(\alpha \right)\left[\frac{d}{d\alpha }\Upsilon \left(\alpha \right)\right]\Upsilon \left(\alpha \right)+\Upsilon {\left(\alpha \right)}^{2}\frac{d}{d\alpha }\Upsilon \left(\alpha \right).$$

By (A28), bearing in mind the that matrices Υ(α) and $\frac{d}{d\alpha }\Upsilon \left(\alpha \right)$ are symmetric, we have

(A29) $${\bar{G}}_{N}^{T}\Upsilon {\left(\alpha \right)}^{-3}\left[\frac{d}{d\alpha }\Upsilon {\left(\alpha \right)}^{3}\right]\Upsilon {\left(\alpha \right)}^{-3}{\bar{G}}_{N}=2{\bar{G}}_{N}^{T}\Upsilon {\left(\alpha \right)}^{-3}\left[\frac{d}{d\alpha }\Upsilon \left(\alpha \right)\right]\Upsilon {\left(\alpha \right)}^{-1}{\bar{G}}_{N}+{\bar{G}}_{N}^{T}\Upsilon {\left(\alpha \right)}^{-2}\left[\frac{d}{d\alpha }\Upsilon \left(\alpha \right)\right]\Upsilon {\left(\alpha \right)}^{-2}{\bar{G}}_{N}.$$

Similarly, by (A27), we obtain

(A30) $${\bar{G}}_{N}^{T}\Upsilon {\left(\alpha \right)}^{-2}\left[\frac{d}{d\alpha }\Upsilon {\left(\alpha \right)}^{2}\right]\Upsilon {\left(\alpha \right)}^{-2}{\bar{G}}_{N}=2{\bar{G}}_{N}^{T}\Upsilon {\left(\alpha \right)}^{-2}\left[\frac{d}{d\alpha }\Upsilon \left(\alpha \right)\right]\Upsilon {\left(\alpha \right)}^{-1}{\bar{G}}_{N}.$$

Including (A29), (A30) and (A27), Equation (A26) is, after algebraic manipulations, rewritten as

$$2{\bar{G}}_{N}^{T}\Upsilon {\left(\alpha \right)}^{-3}\left[\frac{d}{d\alpha }\Upsilon \left(\alpha \right)\right]\Upsilon {\left(\alpha \right)}^{-1}{\bar{G}}_{N}tr\left[\Upsilon {\left(\alpha \right)}^{-1}\right]+{\bar{G}}_{N}^{T}\Upsilon {\left(\alpha \right)}^{-2}\left[\frac{d}{d\alpha }\Upsilon \left(\alpha \right)\right]\Upsilon {\left(\alpha \right)}^{-2}{\bar{G}}_{N}tr\left[\Upsilon {\left(\alpha \right)}^{-1}\right]+\;{\bar{G}}_{N}^{T}\Upsilon {\left(\alpha \right)}^{-3}{\bar{G}}_{N}tr\left[\Upsilon {\left(\alpha \right)}^{-1}\frac{d}{d\alpha }\Upsilon \left(\alpha \right)\Upsilon {\left(\alpha \right)}^{-1}\right]=2{\bar{G}}_{N}^{T}\Upsilon {\left(\alpha \right)}^{-2}\left[\frac{d}{d\alpha }\Upsilon \left(\alpha \right)\right]\Upsilon {\left(\alpha \right)}^{-1}{\bar{G}}_{N}\;tr\left[\Upsilon {\left(\alpha \right)}^{-2}\right]+\;{\bar{G}}_{N}^{T}\Upsilon {\left(\alpha \right)}^{-2}{\bar{G}}_{N}\;tr\left[\left[\Upsilon {\left(\alpha \right)}^{-2}\left[\frac{d}{d\alpha }\Upsilon \left(\alpha \right)\right]\Upsilon {\left(\alpha \right)}^{-1}+\Upsilon {\left(\alpha \right)}^{-1}\left[\frac{d}{d\alpha }\Upsilon \left(\alpha \right)\right]\Upsilon {\left(\alpha \right)}^{-2}\right]\right].$$

By (A23), the last equation can be expressed as follows:

$$2{\bar{G}}_{N}^{T}\Upsilon {\left(\alpha \right)}^{-3}\left[\Omega_{N,K}\left(\alpha \right)+\frac{d\lambda_{GCV}\left(\alpha \right)}{d\alpha }I_{N,N}\right]\Upsilon {\left(\alpha \right)}^{-1}{\bar{G}}_{N}tr\left[\Upsilon {\left(\alpha \right)}^{-1}\right]+{\bar{G}}_{N}^{T}\Upsilon {\left(\alpha \right)}^{-2}[\Omega_{N,K}\left(\alpha \right)+\;\frac{d\lambda_{GCV}\left(\alpha \right)}{d\alpha }I_{N,N}]\Upsilon {\left(\alpha \right)}^{-2}{\bar{G}}_{N}tr\left[\Upsilon {\left(\alpha \right)}^{-1}\right]+{\bar{G}}_{N}^{T}\Upsilon {\left(\alpha \right)}^{-3}{\bar{G}}_{N}tr\left[\Upsilon {\left(\alpha \right)}^{-1}\left[\Omega_{N,K}\left(\alpha \right)+\frac{d\lambda_{GCV}\left(\alpha \right)}{d\alpha }I_{N,N}\right]\Upsilon {\left(\alpha \right)}^{-1}\right]=\;2{\bar{G}}_{N}^{T}\Upsilon {\left(\alpha \right)}^{-2}\left[\Omega_{N,K}\left(\alpha \right)+\frac{d\lambda_{GCV}\left(\alpha \right)}{d\alpha }I_{N,N}\right]\Upsilon {\left(\alpha \right)}^{-1}{\bar{G}}_{N}\;tr\left[\Upsilon {\left(\alpha \right)}^{-2}\right]+{\bar{G}}_{N}^{T}\Upsilon {\left(\alpha \right)}^{-2}{\bar{G}}_{N}\;tr[[\Upsilon {\left(\alpha \right)}^{-2}[\Omega_{N,K}\left(\alpha \right)+\;\frac{d\lambda_{GCV}\left(\alpha \right)}{d\alpha }I_{N,N}]\Upsilon {\left(\alpha \right)}^{-1}+\Upsilon {\left(\alpha \right)}^{-1}\left[\Omega_{N,K}\left(\alpha \right)+\frac{d\lambda_{GCV}\left(\alpha \right)}{d\alpha }I_{N,N}\right]\Upsilon {\left(\alpha \right)}^{-2}]],$$

which, after painstaking algebraic manipulations, yields Equation (41), which completes the proof. □

### Appendix A.7. Proof of Proposition 1

By (6), for any time-scale factor α and any vector of model parameters $g_{K}$, we have

(A31) $${\left\|H_{K}\left(\tau ,\alpha \right)\right\|}_{2}^{2}=\int_{0}^{\infty }{\left[\sum_{k=0}^{K-1}g_{k}h_{k}\left(\tau ,\alpha \right)\right]}^{2}d\tau ,$$

which is rewritten as

(A32) $${\left\|H_{K}\left(\tau ,\alpha \right)\right\|}_{2}^{2}=\sum_{k=0}^{K-1}\sum_{j=0}^{K-1}g_{k}g_{j}\gamma_{kj}\left(\alpha \right).$$

where the functions

(A33) $$\gamma_{kj}\left(\alpha \right)=\int_{0}^{\infty }h_{k}\left(\tau ,\alpha \right)h_{j}\left(\tau ,\alpha \right)d\tau ,$$

for k,j=0,1,…K−1.

By virtue of (A33), we have $\gamma_{kj}\left(\alpha \right)=\gamma_{jk}\left(\alpha \right)$ for any k,j=0,1,…K−1. The proof is based on the following integral formula [64] (Equation (3.351.3)):

(A34) $$\int_{0}^{\infty }\tau^{n}e^{-2\alpha \tau }d\tau =\frac{n!}{{\left(2\alpha \right)}^{n+1}}.$$

For k=j=0, definition (A33) and (4) immediately imply

(A35) $$\gamma_{00}\left(\alpha \right)=\int_{0}^{\infty }e^{-2\alpha \tau }d\tau =\frac{1}{2\alpha }.$$

For k=0 and j=1,…K−1, by virtue of (A33), (3), (4) and (A34), we have

(A36) $$\gamma_{0j}\left(\alpha \right)={\left(\frac{e\alpha }{j}\right)}^{j}\int_{0}^{\infty }\tau^{j}e^{-2\alpha \tau }d\tau ={\left(\frac{e\alpha }{j}\right)}^{j}\frac{j!}{{\left(2\alpha \right)}^{j+1}}=\frac{1}{2\alpha }{\left(\frac{e}{2}\right)}^{j}\frac{j!}{j^{j}}.$$

For k,j=1,…K−1, through (A33), (3) and (A34), we obtain

$$\gamma_{kj}\left(\alpha \right)=e^{k+j}{\left(\frac{\alpha }{k}\right)}^{k}{\left(\frac{\alpha }{j}\right)}^{j}\int_{0}^{\infty }\tau^{k+j}e^{-2\alpha \tau }d\tau =e^{k+j}{\left(\frac{\alpha }{k}\right)}^{k}{\left(\frac{\alpha }{j}\right)}^{j}\frac{\left(k+j\right)!}{{\left(2\alpha \right)}^{k+j+1}}.$$

The last equation is expressed as

(A37) $$\gamma_{kj}\left(\alpha \right)=\frac{1}{2\alpha }{\left(\frac{e}{2}\right)}^{k+j}\frac{\left(k+j\right)!}{k^{k}\;j^{j}}.$$

Bearing in mind (A33), Equation (A32) is rewritten as the quadratic form

(A38) $${\left\|H_{K}\left(\tau ,\alpha \right)\right\|}_{2}^{2}=g_{K}^{T}\Gamma \left(\alpha \right)g_{K},$$

where symmetric K×K matrix $\Gamma \left(\alpha \right)=\left[\gamma_{kj}\left(\alpha \right)\right]$, composed by the functions $\gamma_{kj}\left(\alpha \right)$ defined by (A33), through (A35)–(A37), is as follows

(A39) $$\Gamma \left(\alpha \right)=\frac{1}{2\alpha }\left[\begin{array}{cccccc} 1 & \frac{e}{2} & \cdots & {\left(\frac{e}{2}\right)}^{j}\frac{j!}{j^{j}} & \cdots & {\left(\frac{e}{2}\right)}^{K-1}\frac{\left(K-1\right)!}{{\left(K-1\right)}^{K-1}}\\ \frac{e}{2} & 2{\left(\frac{e}{2}\right)}^{2} & \cdots & {\left(\frac{e}{2}\right)}^{1+j}\frac{\left(1+j\right)!}{\;j^{j}} & \cdots & {\left(\frac{e}{2}\right)}^{K}\frac{\left(K\right)!}{\;{\left(K-1\right)}^{K-1}}\\ \vdots & \vdots & \ddots & \vdots & \ddots & \vdots \\ {\left(\frac{e}{2}\right)}^{k}\frac{k!}{k^{k}} & {\left(\frac{e}{2}\right)}^{k+1}\frac{\left(k+1\right)!}{k^{k}} & \cdots & {\left(\frac{e}{2}\right)}^{k+j}\frac{\left(k+j\right)!}{k^{k}\;j^{j}} & \cdots & {\left(\frac{e}{2}\right)}^{k+K-1}\frac{\left(k+K-1\right)!}{k^{k}\;{\left(K-1\right)}^{K-1}}\\ \vdots & \vdots & \vdots & \vdots & \ddots & \vdots \\ {\left(\frac{e}{2}\right)}^{K-1}\frac{\left(K-1\right)!}{{\left(K-1\right)}^{K-1}} & {\left(\frac{e}{2}\right)}^{K-1+j}\frac{\left(K\right)!}{{\left(K-1\right)}^{K-1}} & \cdots & {\left(\frac{e}{2}\right)}^{K-1+j}\frac{\left(K-1+j\right)!}{{\left(K-1\right)}^{K-1}\;j^{j}} & \cdots & {\left(\frac{e}{2}\right)}^{2K-2}\frac{\left(2K-2\right)!}{{\left(K-1\right)}^{2\left(K-1\right)}} \end{array}\right].$$

Since matrix Γ(α) can be expressed as $\Gamma \left(\alpha \right)=\frac{1}{2\alpha }\Gamma_{1}$, where $\Gamma_{1}$ is given by (53), Equation (52) is derived.

According to (A38) and (A31), the quadratic form $g_{K}^{T}\Gamma \left(\alpha \right)g_{K}$ is expressed as

$$g_{K}^{T}\Gamma \left(\alpha \right)g_{K}=\int_{0}^{\infty }{\left[\sum_{k=0}^{K-1}g_{k}h_{k}\left(\tau ,\alpha \right)\right]}^{2}d\tau .$$

Thus, $g_{K}^{T}\Gamma \left(\alpha \right)g_{K}\geq 0$ for an arbitrary vector $g_{K}$, and $g_{K}^{T}\Gamma \left(\alpha \right)g_{K}=0$, if and only if $\sum_{k=0}^{K-1}g_{k}h_{k}\left(\tau ,\alpha \right)=0$ for almost all τ>0. Since the basis functions $h_{k}\left(\tau ,\alpha \right)$ are independent, the last equality holds, if and only if $g_{k}=0$ for all k=0,1,…,K−1, i.e., only if the vector $g_{K}=0$, which yields the positive definiteness of Γ(α). Whence, $\Gamma_{1}=2\alpha \Gamma \left(\alpha \right)$. is positive definite, too; this finishes the proof. □

### Appendix A.8. Proof of Proposition 4

Since, by (6) and (58), bearing in mind (A32), (A33), (A38) and $\Gamma \left(\alpha \right)=\frac{1}{2\alpha }\Gamma_{1}$, we have

$${\left\|H_{K}\left(\tau ,\alpha \right)-{\tilde{H}}_{K}\left(\tau ,\alpha \right)\right\|}_{2}^{2}=\frac{1}{2\alpha }{\left[{\bar{g}}_{K}^{\lambda }\left(\alpha \right)-g_{K}^{\lambda }\left(\alpha \right)\right]}^{T}\Gamma_{1}\left[{\bar{g}}_{K}^{\lambda }\left(\alpha \right)-g_{K}^{\lambda }\left(\alpha \right)\right],$$

in view of the right inequality in (54), the following estimation holds:

(A40) $${\left\|H_{K}\left(\tau ,\alpha \right)-{\tilde{H}}_{K}\left(\tau ,\alpha \right)\right\|}_{2}^{2}\leq \frac{1}{2\alpha }\sigma_{1}\left(\Gamma_{1})\right\|{\bar{g}}_{K}^{\lambda }\left(\alpha \right)-g_{K}^{\lambda }{\left(\alpha )\right\|}_{2}^{2}.$$

By virtue of (27) and (59), and bearing in mind (48), we obtain

$${\bar{g}}_{K}^{\lambda }\left(\alpha \right)-g_{K}^{\lambda }\left(\alpha \right)=V\left(\alpha \right)\Lambda_{\lambda }\left(\alpha \right)\;U{\left(\alpha \right)}^{T}z_{N},$$

where the orthogonal matrices V(α) and U(α) are defined by SVD (47), and the diagonal matrix $\Lambda_{\lambda }\left(\alpha \right)$ is given by (49). Thus,

$${\left\|{\bar{g}}_{K}^{\lambda }\left(\alpha \right)-g_{K}^{\lambda }\left(\alpha \right)\right\|}_{2}^{2}=tr\left[U\left(\alpha \right)\Lambda_{\lambda }^{T}\left(\alpha \right)\Lambda_{\lambda }\left(\alpha \right)\;U{\left(\alpha \right)}^{T}z_{N}z_{N}^{T}\right],$$

which, using the Schwarz inequality for matrices [57] and the orthogonality of U(α), is estimated as follows:

(A41) $${\left\|{\bar{g}}_{K}^{\lambda }\left(\alpha \right)-g_{K}^{\lambda }\left(\alpha \right)\right\|}_{2}^{2}\leq tr{\left[\Lambda_{\lambda }^{T}\left(\alpha \right)\Lambda_{\lambda }\left(\alpha \right)\Lambda_{\lambda }^{T}\left(\alpha \right)\Lambda_{\lambda }\left(\alpha \right)\right]}^{\frac{1}{2}}\;\;tr{\left[z_{N}z_{N}^{T}z_{N}z_{N}^{T}\right]}^{\frac{1}{2}}.$$

Since

$$\;tr\left[z_{N}z_{N}^{T}z_{N}z_{N}^{T}\right]=z_{N}^{T}z_{N}tr\left[z_{N}z_{N}^{T}\right]={\left[z_{N}^{T}z_{N}\right]}^{2}={\left\|z_{N}\right\|}_{2}^{4},$$

and by the diagonal structure of $\Lambda_{\lambda }\left(\alpha \right)$ (49), we have

$$tr\left[\Lambda_{\lambda }^{T}\left(\alpha \right)\Lambda_{\lambda }\left(\alpha \right)\Lambda_{\lambda }^{T}\left(\alpha \right)\Lambda_{\lambda }\left(\alpha \right)\right]=\sum_{i=1}^{r\left(\alpha \right)}\frac{{\left[\sigma_{i}\left(\alpha \right)\right]}^{4}}{{\left\{{\left[\sigma_{i}\left(\alpha \right)\right]}^{2}+\lambda \right\}}^{4}},$$

inequality (A41) is equivalently rewritten as

(A42) $${\left\|{\bar{g}}_{K}^{\lambda }\left(\alpha \right)-g_{K}^{\lambda }\left(\alpha \right)\right\|}_{2}^{2}\leq {\left[\sum_{i=1}^{r\left(\alpha \right)}\frac{{\left[\sigma_{i}\left(\alpha \right)\right]}^{4}}{{\left\{{\left[\sigma_{i}\left(\alpha \right)\right]}^{2}+\lambda \right\}}^{4}}\right]}^{\frac{1}{2}}{\left\|z_{N}\right\|}_{2}^{2}.$$

Now, estimations (A40) and (A42) yield inequality (60), and the proposition is proved. □

### Appendix A.9. Derivation of the Formula (62)

To derive Equation (62), note that due to (2), the relaxation modulus induced by the spectrum

(A43) $$H\left(\tau \right)=\left[e^{-{\left(\frac{1}{\tau }-m\right)}^{2}/q}\right]/\tau$$

is described by

$$G\left(t\right)=\int_{0}^{\infty }e^{-{\left(\frac{1}{\tau }-m\right)}^{2}/q}\frac{1}{\tau^{2}}e^{-t/\tau }d\tau .$$

Through (2) and (A43), applying the substitution $\frac{1}{\tau }=x$, the above integral is rewritten as

$$G\left(t\right)=-\int_{\infty }^{0}e^{-{\left(x-m\right)}^{2}/q}e^{-tx}dx=\int_{0}^{\infty }e^{-{\left(x-m\right)}^{2}/q}e^{-tx}dx$$

and next, as follows:

(A44) $$G\left(t\right)=e^{\frac{1}{4}t^{2}q-mt}\int_{0}^{\infty }e^{\frac{-{\left(x+\frac{1}{2}tq-m\right)}^{2}}{q}}dx.$$

Applying in the integral (A44) the substitution $\frac{\left(x+\frac{1}{2}tq-m\right)}{\sqrt{q}}=z$, the formula describing relaxation modulus is expressed as

(A45) $$G\left(t\right)=\sqrt{q}\;e^{\frac{1}{4}t^{2}q-mt}\int_{\left(\frac{1}{2}tq-m\right)/\sqrt{q}}^{\infty }e^{-z^{2}}dz=\frac{\sqrt{q\pi }}{2}\;e^{\frac{1}{4}t^{2}q-mt}erfc\left(\frac{\frac{1}{2}tq-m}{\sqrt{q}}\right),$$

where the function erfc(x) is defined by (63). Formula (62) follows directly from the last expression of the right-hand side of (A45), by comparison of (A43) and (61).

## Appendix B

**Table A1:** Ranges of the applicability of the model for various time-scale parameters for K=6,…11.

Time-Scale Factor *α* [s]	Range ^1^ of Relaxation Times *τ_app_*(*α*) [s]	Range ^1^ of Times *t_app_*(*α*) [s]	Range ^1^ of Relaxation Times *τ_app_*(*α*) [s]	Range ^1^ of Times *t_app_*(*α*) [s]
	K=6	K=6	K=7	K=7
0.0001	161,655.35	337,647.7	178,346.044	392,372
0.001	16,165.53	33,764.77	17,834.606	39,238.5
0.01	1616.55	3376.48	1783.462	3923.85
0.1	161.65	337.65	178.348	392.5
1	16.165	33.765	17.836	39.26
10	1.616	3.376	1.784	3.95
100	0.1616	0.337	0.1783	0.392
α [s]	K=8	K=8	K=9	K=9
0.0001	194,529.30	446,719.4	210,306.21	500,804.6
0.001	19,452.93	44,671.94	21,030.621	50,080.46
0.01	1945.29	4467.19	2103.062	5008.046
0.1	194.53	446.72	210.306	500.81
1	19.453	44.672	21.0306	50.08
10	1.945	4.467	2.1031	5.008
100	0.1945	0.447	0.2103	0.501
α [s]	K=10	K=10	K=11	K=11
0.0001	225,748.07	554,697	240,907.43	608,443.6
0.001	22,574.807	55,469.7	24,090.743	60,844.38
0.01	2257.481	5546.97	2409.074	6084.45
0.1	225.748	554.69	240.907	608.46
1	22.5748	55.469	24.0907	60.86
10	2.2575	5.547	2.4091	6.1
100	0.2257	0.555	0.2409	0.608

^1^ The upper bounds $t_{app}\left(\alpha \right)$ (20) and $\tau_{app}\left(\alpha \right)$ (21) of the applicability intervals $\left[0,t_{app}\left(\alpha \right)\right]$ and $\left[0,\tau_{app}\left(\alpha \right)\right]$ are given.

**Table A2:** The square roots of the upper bounds $\psi_{n}\left(\Gamma_{1}\right)$ (56) and the square roots of the largest $\sigma_{1}\left(\Gamma_{1}\right)$ and minimal $\sigma_{min}\left(\Gamma_{1}\right)$ singular values of the matrix $\Gamma_{1}$ (53) for K=4,5, …12 model summands.

K	4	5	6	7	8	9	10	11	12
n	$\sqrt{\psi_{n}\left(\Gamma_{1}\right)}$								
1	3.680199	4.353830	4.974923	5.556319	6.106199	6.630173	7.132299	7.615642	8.082583
2	3.671711	4.330308	4.931579	5.489724	6.013827	6.510086	6.982965	7.435811	7.871217
3	3.667383	4.325690	4.927344	5.485966	6.010365	6.506625	6.979169	7.431342	7.865747
4	3.666555	4.325232	4.927078	5.485799	6.010251	6.506536	6.979081	7.431227	7.865575
5	3.666421	4.325190	4.927062	5.485792	6.010247	6.506534	6.979079	7.431224	7.865568
6	3.666400	4.325186	4.927061	5.485792	6.010247	6.506534	6.979079	7.431223	7.865567
7	3.666397	4.325186	4.927061	5.485792	6.010247	6.506534	6.979079	7.431223	7.865567
8	3.666396	4.325186	4.927061	5.485792	6.010247	6.506534	6.979079	7.431223	7.865567
9	3.666396	4.325186	4.927061	5.485792	6.010247	6.506534	6.979079	7.431223	7.865567
10	3.666396	4.325186	4.927061	5.485792	6.010247	6.506534	6.979079	7.431223	7.865567
$\sqrt{\sigma_{1}\left(\Gamma_{1}\right)}$	3.666396	4.325185	4.927061	5.485792	6.010247	6.506534	6.979079	7.431223	7.865567

**Table A3:** Optimal parameters ${\hat{g}}_{K}={\hat{g}}_{K}^{\lambda_{GCV}}\left(\hat{\alpha }\right)$ (45) of the relaxation spectrum models from Example 1; the elements of the vectors ${\hat{g}}_{K}$ are expressed in [Pa].

${\hat{g}}_{K}$									
K=3	K=4	K=5	K=6	K=7	K=8	K=9	K=10	K=11	K=12
0.50819	−0.32787	−0.31484	−0.28238	−0.26421	−0.26104	−0.26344	−0.25958	−0.25722	−0.24893
0.48916	3.25406	2.44705	1.86341	1.57535	1.44701	1.38846	1.26949	1.18396	1.042852
−0.01177	−5.37283	−1.74791	−0.21483	0.21735	0.33298	0.38151	0.53024	0.62627	0.77959
	3.79955	−1.90531	−1.81625	−1.22415	−0.94252	−0.85336	−0.72742	−0.63340	−0.49513
		3.00974	−0.42686	−0.80274	−0.65867	−0.57137	−0.58067	−0.58715	−0.58709
			2.49772	0.35990	0.00038	0.018141	−0.06714	−0.11806	−0.19722
				1.85736	0.61157	0.39343	0.27044	0.22125	0.12879
					1.25669	0.58048	0.40004	0.34372	0.26284
						0.75498	0.46114	0.34725	0.26954
							0.59592	0.35547	0.25643
								0.46177	0.31367
									0.49927
